# Different amyloid β42 preparations induce different cell death pathways in the model of SH-SY5Y neuroblastoma cells

**DOI:** 10.1186/s11658-024-00657-8

**Published:** 2024-11-17

**Authors:** Alp Yigit Özdemir, Kateřina Hofbauerová, Vladimír Kopecký, Jiří Novotný, Vladimír Rudajev

**Affiliations:** 1 Department of Physiology, Faculty of Sciences, Charles University, Viničná 7, 12844 Prague 2, Czech Republic; 2 Institute of Physics, Faculty of Mathematics and Physics, Charles University, Ke Karlovu 5, 12116 Prague 2, Czech Republic

**Keywords:** Amyloid β42, Alzheimer´s disease, Cell death, Apoptosis, Necroptosis, Reactive oxygen species, GM1

## Abstract

Amyloid β42 (Aβ42) plays a decisive role in the pathology of Alzheimer’s disease. The Aβ42 peptide can aggregate into various supramolecular structures, with oligomers being the most toxic form. However, different Aβ species that cause different effects have been described. Many cell death pathways can be activated in connection with Aβ action, including apoptosis, necroptosis, pyroptosis, oxidative stress, ferroptosis, alterations in mitophagy, autophagy, and endo/lysosomal functions. In this study, we used a model of differentiated SH-SY5Y cells and applied two different Aβ42 preparations for 2 and 4 days. Although we found no difference in the shape and size of Aβ species prepared by two different methods (NaOH or NH_4_OH for Aβ solubilization), we observed strong differences in their effects. Treatment of cells with NaOH-Aβ42 mainly resulted in damage of mitochondrial function and increased production of reactive oxygen species, whereas application of NH_4_OH-Aβ42 induced necroptosis and first steps of apoptosis, but also caused an increase in protective Hsp27. Moreover, the two Aβ42 preparations differed in the mechanism of interaction with the cells, with the effect of NaOH-Aβ42 being dependent on monosialotetrahexosylganglioside (GM1) content, whereas the effect of NH_4_OH-Aβ42 was independent of GM1. This suggests that, although both preparations were similar in size, minor differences in secondary/tertiary structure are likely to strongly influence the resulting processes. Our work reveals, at least in part, one of the possible causes of the inconsistency in the data observed in different studies on Aβ-toxicity pathways.

**Graphical Abstract:**

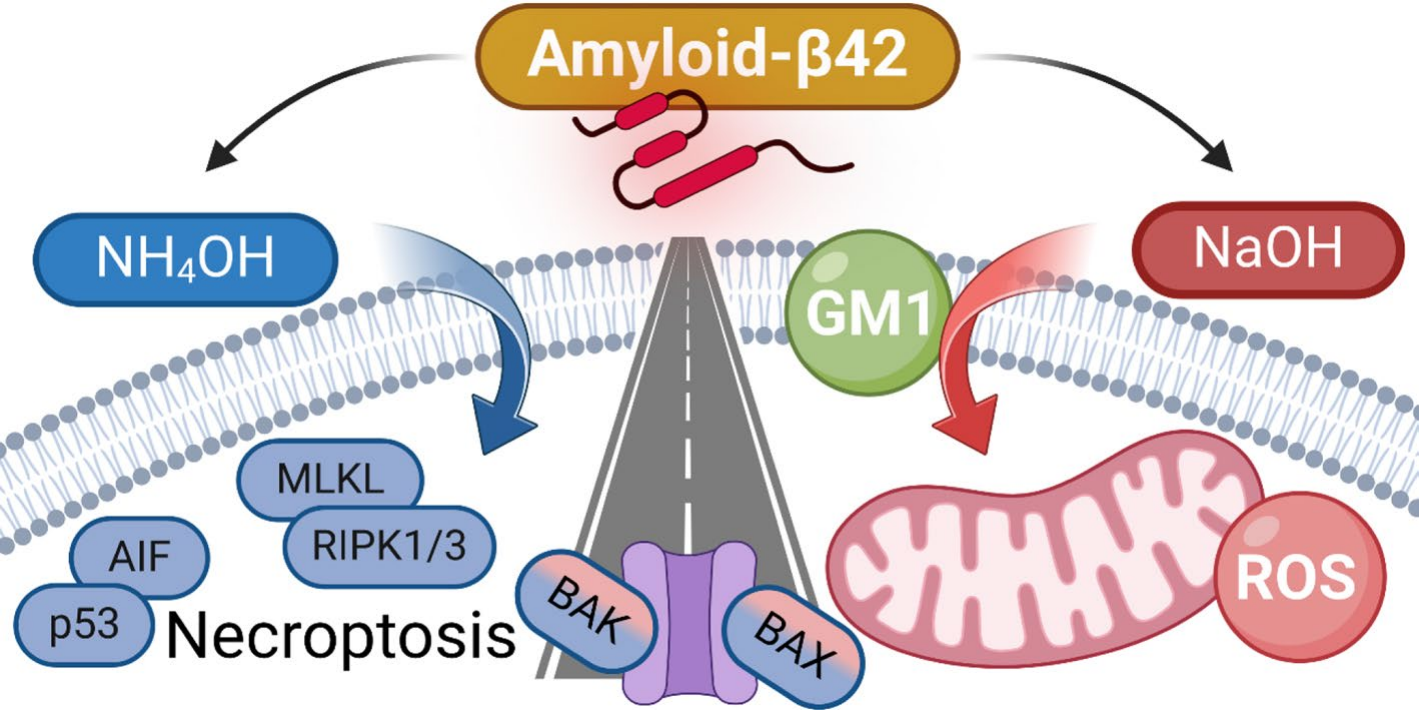

**Supplementary Information:**

The online version contains supplementary material available at 10.1186/s11658-024-00657-8.

## Introduction

Various neuronal death pathways have been described in association with Alzheimer’s disease (AD). Apoptosis, necroptosis, reactive oxygen species (ROS)-induced damage (including ferroptosis), pyroptosis, mitochondrial malfunction, and fatal alterations in autophagy, mitophagy, or lysosomal functioning are induced by amyloid β (Aβ) [1].

Various Aβ species differing in size and shape may induce distinct effects, but Aβ42 oligomers represent the most toxic form [2–6]. Their size is estimated to be about 5–20 nm (> 50 kDa), which corresponds to a composition ranging from a few to tens of monomers [7–13]. Different Aβ aggregates show different behavior that also depends on cell type [4, 14]. Furthermore, Aβ oligomers of similar molecular weight (MW) may differ in secondary, tertiary, and quaternary structure, all of which may affect Aβ–cell interactions and consequential effects connected with toxicity [5, 15, 16]. The ability of Aβ to induce cell death (CD) also depends on membrane composition, where various Aβ receptors including ganglioside GM1 play a role [8, 17–23]. Therefore, the selection of an experimental model and appropriate Aβ species is of principal importance, and it is not surprising that activation of different CD pathways has been described in association with Aβ action. In the following section, we briefly describe the CD pathways that are related to AD.

*Apoptosis*: Some extracellular signals induce extrinsic apoptosis by activation of caspase 8 (Cas8) within the membrane death-inducing signalling complex. Activation of executioner Cas3 follows, which is an element common to both extrinsic and intrinsic apoptosis (reviewed in Refs. [24–26]). Intrinsic apoptosis is programmed CD initiated by various signals associated with enhanced ROS production, damage of DNA, endoplasmic reticulum (ER) stress, Ca^2+^ elevation, etc. The mitochondrion stays at the center of events: the proapoptotic inputs cause permeabilization of mitochondrial membranes, which leads to alteration in the inner membrane potential, decreased ATP production, and release of Ca^2+^ ions. Under cellular stress, BAK and BAX are released from antiapoptotic prosurvival proteins (e.g., Bcl-XL, Bcl-2) and self-organize into the mitochondrial outer membrane pore (MOMP). Several factors are then released from mitochondria, including cytochrome c and apoptosis-inducing factor (AIF), which may lead to DNA damage and activation of Cas9 and executioner Cas3/7 [24, 26, 27]. Activated caspases cleave cellular proteins, leading to disruption of the plasma membrane (PM) and extracellular exposure of phosphatidylserine and CD. Caspase-independent apoptosis includes MOMP formation, DNA cleavage, and enhanced ROS production, but no activity of caspases [1, 28–32].

*ROS-induced CD and ferroptosis*: In ferroptosis, elevated iron levels increase the production of ROS, which cause lipid peroxidation, decreased functionality of antioxidant systems, and severe mitochondrial damage, all of which are associated with AD [33–38]. However, even Aβ42 alone may induce ROS generation and CD, as shown in various models, including neuroblastoma SH-SY5Y cells [34, 39–44].

*Necroptosis* represents a programmed form of necrosis associated with organization of a necrosome—a complex composed of receptor-interacting protein kinases (RIPK) 1 and 3 and mixed lineage kinase domain-like pseudokinase (MLKL), whose activation by phosphorylation and oligomerization leads to permeabilization of PM and CD [26, 45–48]. *Pyroptosis* is associated with inflammation and thus strongly related to AD [26, 49]. The key structure is the inflammasome that can activate Cas1/11. Cas1 then cleavages gasdermin D, whose fragments create a pore in PM and cause necrosis (reviewed in Refs. [1, 50]).

In AD, often following Aβ action, the cellular degradative apparatuses are damaged at different levels and in many ways. *Lysosomal dysfunction* reflects the aggregation of Aβ, which induces membrane permeabilization, H^+^ leakage, release of cathepsins, accumulation of damaged proteins and mitochondria, and thus increased ROS production [1, 26, 51, 52]. Autophagy and mitophagy serve as important protective mechanisms, hence their disruption may also lead to CD. Both decreased and increased autophagy/mitophagy are connected with AD-related CD as a consequence of nonsufficient degradation of misfolded and aggregated proteins, or hyperactivated degradation of cytoplasmic components [26, 53–57].

The pathways of CD, including apoptosis, autophagy, mitophagy, or necroptosis, cross-talk directly or indirectly at multiple levels, complicating the ability to characterize specific processes [1, 48, 58–63]. Caspases may inhibit autophagy or necroptosis [27], and Cas8 or RIPK1 play roles in extrinsic and intrinsic apoptosis as well as necroptosis or inflammation [24–26, 31, 48, 64–66]. Aβ-induced apoptosis may affect inflammasome activation, but also inflammasome protein NLRP3 can activate Cas8 and induce apoptosis [16, 67]. Furthermore, by elevating ROS or cytosolic Ca^2+^ concentration, Aβ impairs many cellular functions [19, 68, 69]. Time course and subcellular localization of events, as well as a level of stress, also determine the direction toward a particular CD [27, 58–61, 70, 71]. Finally, several processes may occur simultaneously, as was shown in rats, where necroptosis and ferroptosis were activated, but also Cas8 was upregulated. There, ferroptosis was upstream of necroptosis, which indicates the complexity of Aβ effects on cell survival [72]. All interactions between CD pathways strongly depend on cellular context as well as on the Aβ species and the selected model. Also, some types of CD may represent attractors for different entries; e.g., DNA damage, ROS elevation, ER stress, energy deficiency, mitochondrial damage, and some extrinsic imputes may all result in apoptosis.

In the present study, we focused on the characterization of the CD pathways induced by two distinct Aβ42 oligomeric aggregates, viz. NaOH-Aβ42 and NH_4_OH-Aβ42 preparations. We used a model of differentiated neuroblastoma SH-SY5Y cells and tried to describe the effects elicited by distinct Aβ oligomers. We found significant differences at several levels that emphasize the complexity of Aβ action and its dependence on the precise character of the Aβ species.

## Materials and methods

### Materials

Amyloid beta 1–42 (CAS no. 107761-42-2) was obtained from Bachem AG (Switzerland). Trypan-Blue (CAS no. 72-57-1), Dulbecco’s modified Eagle’s medium—high glucose (D6429-500 ml), retinoic acid (CAS Number: 302-79-4), antibiotic antimycotic solution (100 ×), 1,1,1,3,3,3-hexafluoro-2-propanol (HFIP) (CAS no. 920-66-1), stabilized A5955, Acridine Orange (AO) hemi(zinc chloride) salt (CAS no. 10127–02-3), propidium iodide (PI) solution (CAS no. 25535-16-4), 2′,7′-dichlorodihydrofluorescein diacetate (DCF-DA) (CAS no. 2044-85-1), malondialdehyde bis(dimethyl acetal) (CAS no. 102–52-3), trichloroacetic acid (TCA) (CAS no. 76-03-9), 2-thiobarbituric acid (TBA) (CAS no. 504-17-6), cholera toxin B subunit (C3741), dimethyl sulfoxide (DMSO) (CAS no. 67–68-5), trypsin–EDTA solution (T4174), sodium hydroxide (CAS no. 1310-73-2), ammonia solution 28–30% (CAS no. 1336-21-6), Ponceau S (CAS no. 6226-79-5), ammonium persulfate (APS) (CAS no. 7727-54-0), Complete™, Mini Protease Inhibitor Cocktail (11,836,153,001), PhosSTOP (PHOSS-RO), sodium carbonate (CAS no. 497-19-8), glycerol (CAS no. 56-81-5), sodium tartrate dihydrate (CAS no. 6106-24-7), copper(II) sulfate pentahydrate (CAS no. 7758-99-8), ethanol (CAS no. 64-17-5), sodium chloride (CAS no. 7647-14-5), bovine serum albumin (BSA) (CAS no. 9048-46-8), anti-amyloid-β (oligomer) antibody, clone F11G3 antibody (MABN1839), and thiazolyl blue tetrazolium bromide (CAS no. 298-93-1) were purchased from Merck–Sigma-Aldrich (Germany). BAK (sc-517390), caspase 3 (sc-7148), caspase 12 (sc-5627), AIF (sc-13116), ATF6α (sc-166659), SOD-2 (sc-30080), F_1_-ATPase (sc-55597), spectrin α II (sc-48382), HSP70 (sc-1060), HSP90 (sc-13119), HSP27 (sc-101699), MLKL (sc-293201), RIP3 (sc-374639), β-amyloid antibody (B-4) (sc-28365), rabbit anti-mouse IgG-HRP (sc-358914), mouse anti-rabbit IgG-HRP (sc-2357), and mouse anti-goat IgG-HRP (sc-2354) antibodies were purchased from Santa Cruz Biotechnology (Germany). BAX (ab32503), p53 (A19585), mt-TFA (ab131607), gasdermin D, and phospho-MLKL-T357/S358/S360 (AP0949) antibodies were purchased from ABclonal (Germany). Glycine (CAS no. 56–40-6), Tween^®^ 20 (CAS no. 9005-64-5), bromophenol blue·Na salt (CAS no. 34725-61-6), acrylamide/bis Solution (10,688.01), and Tris(hydroxymethyl)aminomethane (CAS no. 77-86-1) were obtained from SERVA (Germany). Fetal bovine serum (cat. no. 10270106), Hoechst 33,258, pentahydrate (bis-benzimide) (cat. no. H3569), MitoTracker™ dyes for mitochondria labeling (cat. no. M7514), MitoTracker^™^ dyes for mitochondria labeling (cat. no. M7512), SuperSignal™ West Femto maximum sensitivity substrate (cat. no. 34095), and TEMED (N,N,N’,N’-tetramethyl-ethylenediamine) (cat. no. 17919) were purchased from ThermoFisher Scientific (Germany). Annexin V binding buffer (10×) and Annexin V conjugated to Dyomix 647 were purchased from Exbio (Czechia). Premium Western blotting membranes were purchased from Amersham^™^ Protran^®^ (NJ, USA). Phospho-RIP3 (Ser232) antibody (#AF7443) was purchased from SVEN BioLabs s.r.o. (Czechia). PARP1 polyclonal antibody (cat. no. E-AB-16025) was purchased from Elabscience (USA).

### Cell culture and differentiation of SH-SY5Y cells

Human neuroblastoma SH-SY5Y cells (ATCC^®^ CRL-2266^™^) were incubated in the Dulbecco’s modified Eagle’s medium—high glucose (DMEM-HG) with 10% fetal bovine serum (FBS) and 1% 100× penicillin/streptomycin (P/S) before differentiation. Then, they differentiated for 5–11 days in the differentiation medium (DMEM-HG, 1% FBS, 1% P/S, 100 μM retinoic acid). After differentiation, the expression levels of synaptophysin (Syp) and tyrosine hydroxylase (TH), which are expressed in mature neurons, increased. The differentiated cells were treated with different concentrations of Aβ oligomers for 2 or 4 days, and, in the case of GM1 manipulation, we applied ( +)-d-threo-PDMP (*N*-[(1*R*,2*R*)-2-hydroxy-1-(4-morpholinylmethyl)-2-phenylethyl]-decanamide, D-PDMP) (Cayman Chemical, MI, USA).

### Amyloid-β oligomerization

Aβ was oligomerized using NaOH and NH_4_OH as described before [73–76]. Initially, Aβ was dissolved in 1,1,1,3,3,3-hexafluoro-2-propanol (HFIP). HFIP is widely used as an agent that solubilizes Aβ into monomers by disrupting noncovalent interaction between Aβ peptides. After HFIP solubilization, the samples were subjected to overnight evaporation. Aliquots of 100 µg were frozen at −20 °C. Before application, the HFIP solubilization was repeated and a 1-h vacuum evaporation step was added. The resulting pellets were dissolved in either 10 mM NaOH or 10% NH_4_OH (2, 85 M) solution. After another drying process, the pellets were frozen (−20 °C) for later use or dissolved in 2 µl dimethyl sulfoxide (DMSO) and then diluted with phosphate-buffered saline (PBS) (137 mM NaCl, 2.7 mM KCl, 10 mM Na_2_HPO_4_, 1.8 mM KH_2_PO_4_, pH 7.4) toa final concentration of 1 µg/µl (451 µM). Aβ in PBS was frozen (−20 °C, for a maximum of 1 month) or used immediately.

### Dynamic light scattering

Dynamic light scattering (DLS) measurements were performed at a constant temperature of 20 °C on a Zetasizer Nano ZS (Malvern Instruments, UK) equipped with a He–Ne laser with a wavelength of 633 nm. Protein samples (dissolved in PBS buffer, 1 mg/ml) were centrifuged for 5 min, 20,000 revolutions, cooling at 10 °C to remove dust particles before measurement. Aβ42 were measured in a 40 µL 3 × 3 mm quartz cuvette recommended by the manufacturer (Hellma Analytics, Germany). Each sample was measured in two series of ten runs. The resulting DLS data are given as averages of individual investigated values. The results were processed using the original Zetasizer 6.2 software (Malvern Instruments, Great Britain).

### Atomic force microscopy

Aβ42 samples were dissolved in PBS buffer at a concentration of 0.01 mg/ml. Peptide–mica samples for atomic force microscopy (AFM) were prepared in the standard following way [77]: Solutions of amyloid were adsorbed on fleshly cleaved muscovite mica (V1 quality, Electron Microscopy Sciences, USA) for 20 min. Then, the excessive liquid sample was removed and the mica was heated up to 60 °C to avoid the formation of the coffee ring effect [78, 79] and pattern formation [80] and subsequent high-density induced fibrils formation. Finally, the mica was completely rinsed with ultrapure water and rapidly dried at 60 °C. AFM imaging was performed in contact mode with combined Raman/AFM/SNOM micro-spectrometer alpha300 RSA (WITec, Germany) using silicon AFM Arrow cantilevers (0.2 N/m, 14 kHz, WITec, Germany). The data were subsequently analyzed and images were treated using Project SIX Plus 6.0 software (WITec, Germany).

### Determination of cell viability

Cell viability was measured with the 3-(4,5-dimethylthiazol-2-yl)-2,5-diphenyltetrazolium bromide (MTT) and Trypan blue (TB) assays. The MTT test is a calorimetric cell viability assay that determines the number of living cells by analyzing the formazan crystals generated by viable cells [81]. Cells were seeded to 96-well plates at a density of 7500 cells/well for differentiation and treatment. For the MTT assay, as detailed in earlier studies [81, 82], cells were washed with PBS after removing the culture medium. Cells were incubated with a serum-free medium containing 10% MTT solution, for 4 h at 37 °C with 5% CO_2_. DMSO was used to dissolve the formazan crystals, creating a violet-like color. Absorbance at 570 nm was measured using a Biotek Synergy HT multi-mode microplate reader (Marshall Scientific, NH, USA). The percentage of cell viability compared with the control group was determined using the measured absorbance values and compared statistically with each other.

TB is a dye that selectively stains dead cells. Healthy cells possess intact cell membranes, which prevent the dye from entering the cell. In contrast, damaged or dead cells have compromised membranes, allowing TB to penetrate and stain them [83, 84]. Cells were seeded to 25 cm^2^ flasks at a density of 2 × 10^6^ cells/flask. After the treatment, cells were washed with PBS, trypsinised, and centrifuged. The resulting cell pellet was then resuspended in PBS and incubated at room temperature with 0.4% TB dye solution for 5 min. Subsequently, the mixture was examined under a microscope, and both viable (unstained) and nonviable (stained) cells were counted to assess cell viability accurately.

### Assessment of oxidative stress and mitochondrial damage

The DCF-DA assay was employed to quantify the level of ROS and oxidative stress [42]. This assay relies on the diffusion of DCF-DA into cells, where cellular esterases catalyze its deacetylation, yielding a nonfluorescent molecule. Upon oxidation by ROS, this molecule is converted to 2′,7′-dichlorofluorescein (DCF), a fluorescent compound. The fluorescence of DCF can be detected and quantified using fluorescence spectroscopy at wavelengths of 485 nm for excitation and 535 nm for emission [85]. Cells were seeded on 96-well plates and, after Aβ treatment, incubated with DCF-DA solution for 30 min at 37 °C/5% CO_2_. Following the incubation period, the DCF-DA solution was removed, and the cells were further washed with PBS. Imaging was performed using an inverted fluorescence microscope AIF 5114i-T (ARSENAL, Czechia). The acquired images were analyzed using ImageJ software (National Institutes of Health, USA).

The thiobarbituric acid reactive substances (TBARS) assay is based on the principle of measuring the end-products of lipid peroxidation, primarily malondialdehyde (MDA) and related substances. Under acidic and high-temperature conditions, MDA reacts with thiobarbituric acid (TBA) to form a pink-colored complex. The intensity of this color is proportional to the concentration of MDA in the sample and was measured spectrophotometrically at around 532–535 nm. Calibration with known MDA standards allows for the quantification of lipid peroxidation, providing a valuable tool to assess oxidative damage to lipids in biological samples [85]. To investigate the concentration of malondialdehyde (MDA), cells were harvested into an ice-cold homogenizing buffer and proteins were extracted. Subsequently, the collected proteins underwent treatment with thiobarbituric acid (TBA) and trichloroacetic acid (TCA). The MDA levels in the samples were then quantified using a Biotek Synergy HT multi-mode microplate reader (Marshall Scientific, NH, USA) with fluorescence excitation at 540/25 nm and emission at 590/35 nm. Statistical methods were employed to compare the measured MDA levels among the different samples.

Mitotracker Green is a selective probe that labels mitochondria by binding to mitochondrial proteins and aggregating in the mitochondrial matrix. Importantly, the reaction of Mitotracker Green remains unaffected by changes in the mitochondrial membrane potential. This feature makes it a valuable tool for assessing mitochondrial mass, as it provides a reliable measure regardless of variations in membrane potential [87, 88]. MitoTracker Red, however, is a fluorescent dye that specifically targets mitochondria and is used to measure mitochondrial membrane potential. This dye can only enter mitochondria with a negatively charged and polarized inner membrane, which is indicative of proper mitochondrial function. Thus, MitoTracker Red serves as a useful agent for assessing mitochondrial health and energy production [88, 89].

To assess mitochondrial mass and membrane potential, as described before ([90]), cells were washed with PBS after removing the culture medium and incubated with either 20 nM MitoTracker Green or 20 nM MitoTracker Red. After the incubation, cells were trypsinized and analyzed using flow cytometry with 488 ex 525/50 bp filter for Mitotracker Green and 561 ex 586/15 bp filter for Mitotracker Red by LSRII flow cytometer (BD Biosciences, USA). All obtained data were processed using Kaluza Analysis 2.1 software (Beckman Coulter, USA).

### Determination of the extent of apoptosis and necrosis

The Acridine Orange (AO) and propidium iodide (PI) assay is a widely used method to distinguish between apoptotic and necrotic cells on the basis of changes in nuclear morphology and membrane integrity. AO, a nucleic acid-specific dye, can penetrate the membranes of both living and early apoptotic cells, causing their nuclei to emit bright green fluorescence with intact structures. In contrast, PI is impermeable to intact cell membranes but can penetrate the damaged membranes of late apoptotic and necrotic cells, causing their nuclei to emit red fluorescence. Therefore, uniform green fluorescence characterizes living cells with an organized structure. Early apoptotic cells are distinguished by bright-green areas of chromatin condensation, while late apoptotic cells exhibit dense orange chromatin condensation. Necrotic cells are identified by their orange fluorescence and intact nuclei. By using a fluorescence microscope, the AO/PI assay enables the quantification of apoptotic and necrotic cells [91–93]. After cultivation in 25 cm^2^ flasks, the cell culture was washed with PBS and the cells were stained with a 1:1 mixture of AO (final concentration: 0.5 mg/1 mg) and PI (final concentration: 0.5 mg/1 mg) for 1 min. After removing the dye, cells were washed twice with PBS. Imaging was conducted using an inverted fluorescence microscope AIF 5114i-T (ARSENAL, Czechia) and apoptotic and necrotic cells were quantified using ImageJ (National Institutes of Health, USA).

Annexin V is an agent that selectively binds to phosphatidylserine (PS), a membrane lipid exposed on the outer leaflet of apoptotic cell membranes. Concurrently, Hoechst 33,258, a DNA-binding dye, stains the nuclei of cells, aiding in the identification of morphological changes associated with apoptosis and necrosis. By utilizing Hoechst in combination with Annexin V, this staining approach allows for the distinction between cells undergoing apoptosis, where Annexin V binds to PS, and those with disrupted cell membranes characteristic of necrosis. The combined use of Annexin V and Hoechst enhances the specificity of detecting different cell death modalities [90, 94–96].

As described in a previous study [90], cells were harvested and centrifuged. After centrifugation, cells were resuspended in annexin binding buffer (ABB), and Annexin V conjugated to Dyomix 647 was added at a final concentration of 1%. The cells were then incubated on ice in the dark for 30 min. After incubation, cells were centrifugated again and resuspended in 150 μL of ABB. Ten minutes before measurement, Hoechst 33,258 was added to the cells at a final concentration of 1 μg/mL. Flow cytometric analysis was conducted on an LSRII flow cytometer (BD Biosciences, USA) using the recommended optical configurations. Annexin V–Dyomix 647 was detected using the 640 ex 670/30 bp filter, while Hoechst was measured using the 405 ex 450/40 bp filter. Data obtained from the FC data were processed using Kaluza Analysis 2.1 software (Beckman Coulter, USA).

### Western blotting

The analysis of protein levels after treatment was conducted using Western blotting. As described before [85, 97], cells were harvested, centrifugated, and resuspended in TMES buffer containing a Complete Protease Inhibitor Cocktail. The cell suspension was subjected to 2 × 10 s, 50% intensity sonication using a Bandelin Sonopuls HD 2070 ultrasonic homogenizer (BANDELIN electronic, Germany) for lysis. Samples for detection of phosphorylated RIPK3 and MLKL were lysed using radioimmunoprecipitation assay (RIPA) buffer in the presence of a phosphatase inhibitor (PhosSTOP). The protein separation was done with electrophoresis using 10–15% separation sodium dodecyl sulfate (SDS)-polyacrylamide gel electrophoresis (PAGE) gel. For each sample, 1 μg/μl protein in Laemmli buffer was loaded (20 μg per lane) and separated by size using an electrophoresis apparatus (Bio-Rad Laboratories, Hercules, CA, USA) at 200 V for 45 min. After separation, proteins were subsequently transferred onto nitrocellulose membranes. Nonspecific binding sites were blocked with 5% dry milk dissolved in TBS-Tween buffer. The target proteins were identified with specific antibodies and visualized with the HRP-labelled secondary antibody using the West Femto maximum sensitivity substrate. The resulting images were analyzed using ImageJ and Image Lab, and the data were normalized by β-actin protein expression.

### Statistics

The statistical comparisons were conducted among the results obtained from 3–6 independent experiments. The comparison was performed using one-way analysis of variance (ANOVA), followed by the Tukey post test. The observed differences were determined based on their significance levels determined by *p*-values.

## Results

### Preparation of Aβ42 oligomers

**Fig. 1:**
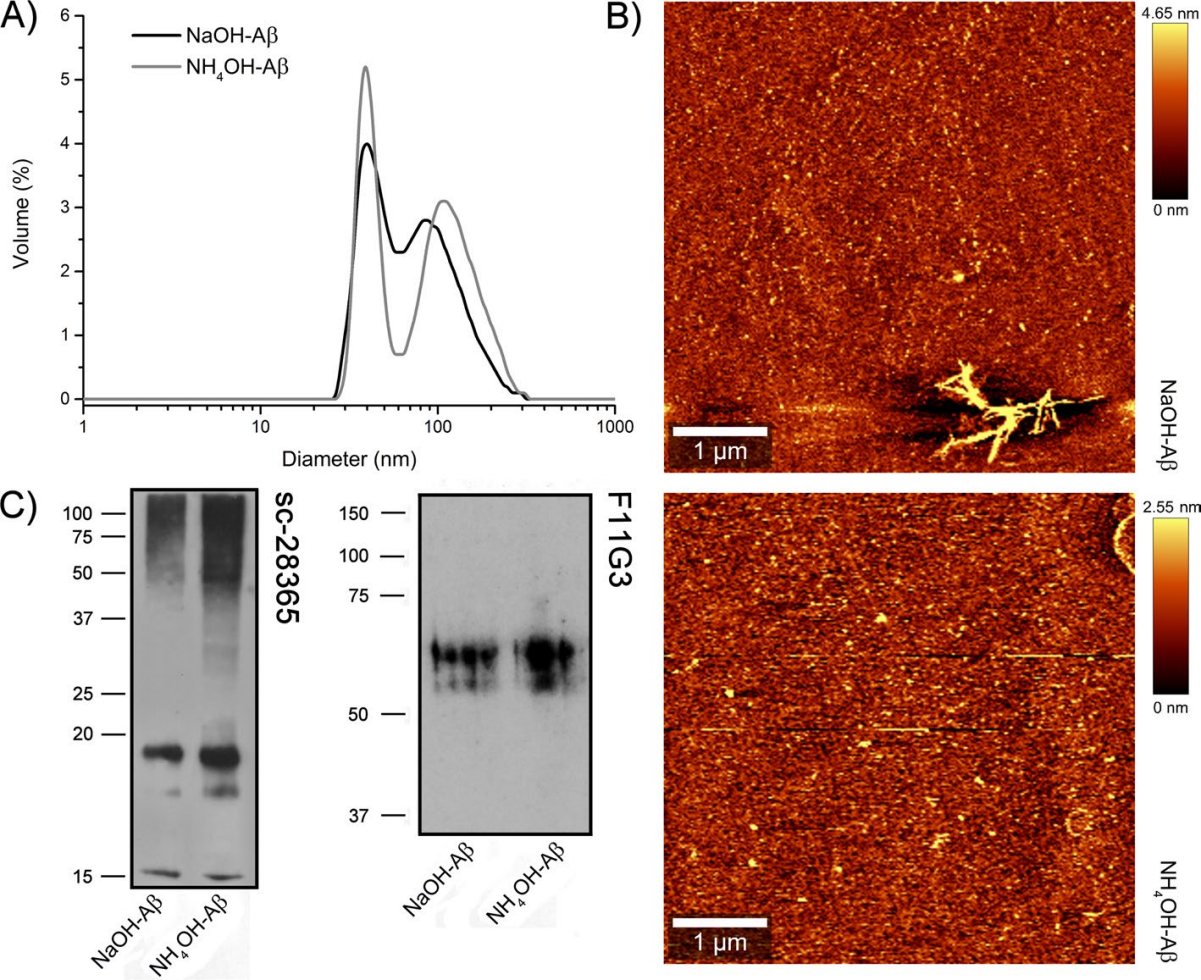
Structural and size characterization of Aβ42 oligomers by (**A**) dynamic light scattering, (**B**) atomic force microscopy, and **C**) western blotting using antibodies against Aβ42. **A** Particle size distribution of NaOH-Aβ42 and NH_4_OH-Aβ42 preparation aggregates given by their volumes (amounts) derived from intensity measurements of DLS. **B** Representative figures from AFM depicting 5 × 5 µm regions where dominantly small globular particles are observable in both NaOH-Aβ42 (above) and NH_4_OH-Aβ42 preparations. Slightly bigger particles are rarely observable as well. Clusters of growing Aβ42 fibrils are observable in bottom left/top right for NaOH-Aβ42/NH_4_OH-Aβ42 preparations. (Such fibrils were removed from the samples by filtration). **C** Western blots of Aβ42 oligomers. 10 µg of NaOH-Aβ42 or NH_4_OH-Aβ42 preparation were loaded on SDS-PAGE. Two different antibodies against Aβ42 were used. Sc-28365 antibodies recognized different amyloid aggregates with strong band corresponding to Aβ42 tetramer (< 20 kDa) and weaker band corresponding to the trimer (~ 15 kDa). The use of antibodies F11G3 developed against Aβ oligomers showed only two bands with MW of 64 and 57 kDa corresponding to ~ 12/14-mers

We prepared Aβ42 oligomers by dissolving the dried peptide in HFIP (see “Methods”). As mainly large amyloid aggregates (measured by DLS) were obtained, we repeated the HFIP solubilization with vacuum evaporation step, and then we solubilized the pellet with agents preventing Aβ aggregation, viz. NaOH or NH_4_OH, respectively [73–76]. DLS measurements indicated that NaOH-Aβ42 samples were somewhat smaller than NH_4_OH-Aβ42 preparations; however, later experiments did not confirm significant size differences (Fig. 1A). On the contrary, there was a dominant representation of smaller oligomers (30–50 nm, with average size 41 nm) in the NH_4_OH-Aβ42 samples, but the majority of the scattered signal corresponded to less populated larger particle sizes of 80–110 nm in both preparations. We thus suppose that the majority of oligomers were composed of > 10 monomers (based on dimension given by Ref. [98]). However, based on DLS spectra, we were not able to distinguish the exact shape, and whether globular or fibrillar oligomers prevail.

In the following step, we used AFM for closer approximation (Fig. 1B). In a general view, it can be stated that both types of samples showed a dominant proportion of small parts of globular shape. The NH_4_OH-Aβ42 aggregates appeared a little higher, which could be due to a greater accumulation of amyloid subunits. In our study, the average height of the aggregates was 4 nm, and their width ranged from roughly 60 to 80 nm, which corresponds to the organization of maximally 20 amyloid peptides in a globular formation (compared with dimensions given in Ref. [98]). However, the detected size will be systematically larger because we did not use high-resolution AFM. This was also confirmed by the comparison with DLS, because the width of the size interval was preserved; only a shift to larger dimensions has occurred. So, we can assume that the size of Aβ42 will be slightly smaller, corresponding to DLS.

Both AFM and DLS did not show a marked size difference between the NaOH-Aβ42 and NH_4_OH-Aβ42 preparations. However, we cannot exclude that both populations differed in the representation profile of the oligomers. For a more detailed view, we analyzed NaOH-Aβ42 and NH_4_-Aβ42 aggregates using western blotting. We used two different antibodies against Aβ42, and none of them showed a marked difference in both NaOH-Aβ42 and NH_4_-Aβ42 preparations. As shown in Fig. 1C, antibodies directed against Aβ42 (sc-28365) gave identical signals corresponding mainly to the tetramer (below 20 kDa) but also to the probable trimer (15 kDa). Because we also obtained an equivocal signal in the region corresponding to MW above 50 kDa, we used specific antibodies against the oligomeric form of Aβ. It can be seen from the figure that these antibodies distinguished two clear bands, the stronger one corresponding to MW of 64 kDa, and the weaker one to 57 kDa. These aggregates thus represented amyloid species composed of ~ 12/14 monomers, which is in accordance with data from DLS and AFM.

### Amyloid β42 toxicity

The SH-SY5Y cell line is a human neuroblastoma cell line that can be differentiated into neuron-like cells, making them suitable for studying the mechanisms of neurodegenerative disorders and testing potential therapeutic interventions [76, 84, 99–101]. We used differentiated SH-SY5Y cells as a model for Aβ42 toxicity. Differentiation was achieved for 7 days by using 100 μM retinoic acid (see “Methods”) and was verified by measuring protein markers (synaptophysin, tyrosine hydroxylase) on immunoblot (Supplementary Fig. S1).

**Fig. 2:**
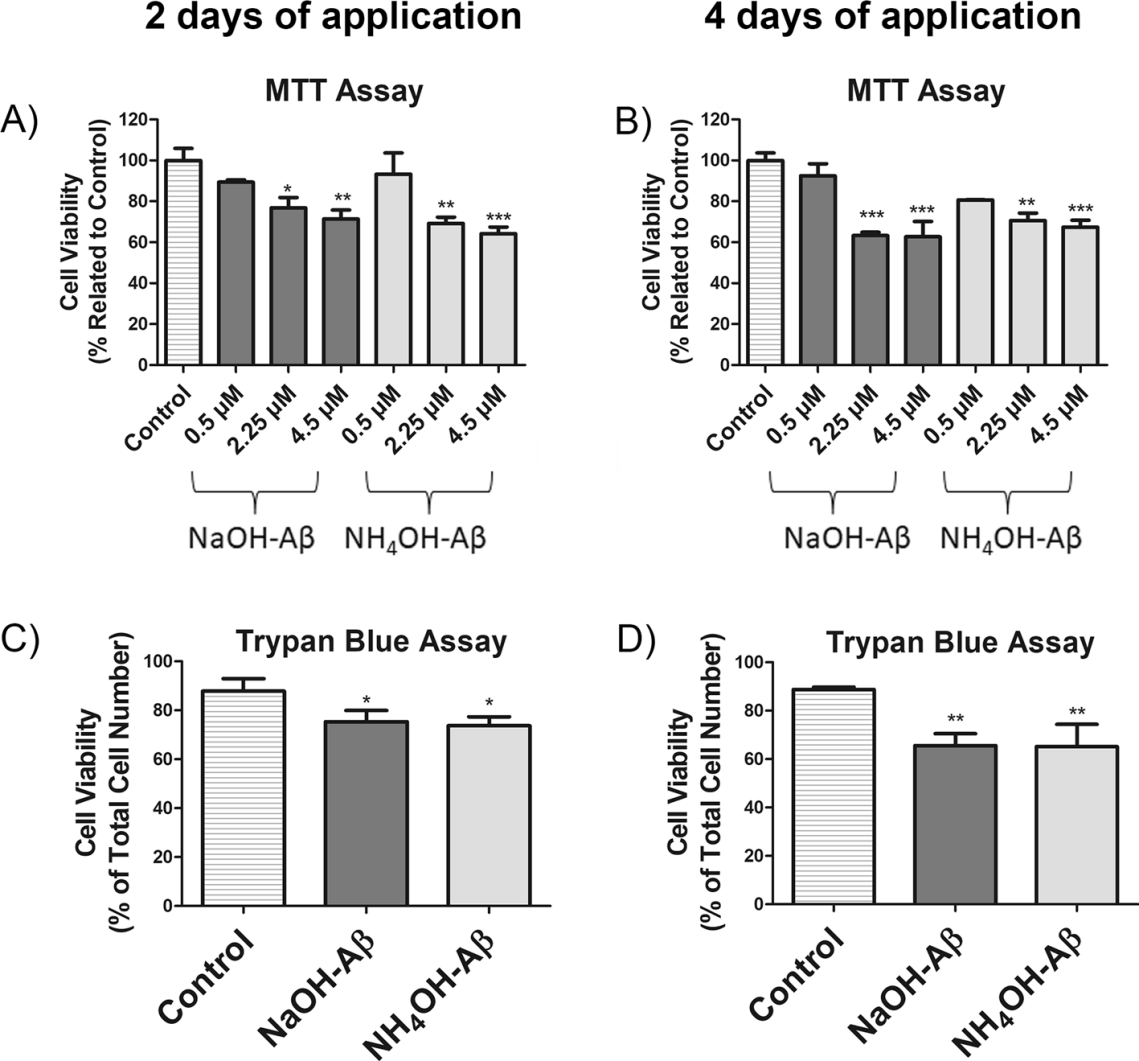
Cell viability assessment. Quantitative assessment of cellular viability following treatment with NaOH and NH_4_OH-Aβ42 applications via MTT (**A**, **B**) and TB (**C**, **D**) assays. **A** MTT analysis after 2 days revealed a significant decrease in viability with both 2.25 μM NaOH-Aβ42 (*n* = 3, 76.78%, *p* = 0.052) and NH_4_OH-Aβ42 (*n* = 3, 69.15%, *p* = 0.007), as well as with 4.5 μM NaOH-Aβ42 (*n* = 3, 71.37%, *p* = 0.013) and NH_4_OH-Aβ42 (*n* = 3, 64.19%, *p* = 0.002). **B** MTT analysis after 4 days showed a further decrease in viability with 2.25 μM NaOH-Aβ42 (*n* = 4, 63.22%, *p* = 0.0002) and NH_4_OH-Aβ42 (*n* = 4, 70.60%, *p* = 0.0019), or with 4.5 μM NaOH-Aβ42 (*n* = 4, 62.67%, *p* = 0.002) and NH_4_OH-Aβ42 (*n* = 4, 67.35%, *p* = 0.0007). The TB assay confirmed reduced viability after 2 days (**C**) with 2.25 μM NaOH-Aβ42 (*n* = 3, 75.35%, *p* = 0.033) and NH_4_OH-Aβ42 (*n* = 3, 73.67%, *p* = 0.019) or after 4 days (**D**) with 2.25 μM NaOH-Aβ42 (*n* = 3, 65.64%, *p* = 0.019) and NH_4_OH-Aβ42 (*n* = 3, 65.19%, *p* = 0.007). Statistical significance level versus untreated samples: *p ≤ 0.05, **p ≤ 0.01, ***p ≤ 0.001

After incubation with Aβ42, we monitored toxicity toward differentiated SH-SY5Y cells by the MTT test, which is widely used for measurement of cell viability [39, 41, 54, 75, 102–104]. We applied three different concentrations of both NaOH-Aβ42 and NH_4_OH-Aβ42 preparations. Figure 2A shows that both types of Aβ42 aggregates reduced the viability of SH-SY5Y cells to below 75% at concentrations of 2.25 and 4.5 µM. We chose the 2,25 µM Aβ42 as a working concentration because it was the lowest concentration with a significant toxic effect.

Incubations with Aβ longer than 2 days are relatively rare [84, 100], while short-term treatments (hours to 2 days) are more common [16, 37, 39, 41, 44, 54, 69, 75, 102, 103, 105]. However, the processes induced by the Aβ are dynamic and may change over time. Therefore, in our model of differentiated SH-SY5Y cells, we compared 2- and 4-day applications of Aβ42 preparations. As shown in Fig. 2B, after 4 days of Aβ treatment, the toxic effect of the amyloid measured by the MTT test was accentuated (Fig. 2B).

The TB assay, which is used for quantifying dead cells [83, 84], confirmed our results with 2.25 µM Aβ obtained by MTT test. The percentage of viable cells in groups treated with NaOH-Aβ42 and NH_4_OH-Aβ42 was determined to be 75.35% and 73.67% after 2 days of application, and 65.64% and 65.19% after 4 days of application, respectively (Fig. 2C, D). We also excluded the toxicity of DMSO and NH_4_OH applied without Aβ (Supplementary Fig. S2).

### Amyloid β42-induced cell death: necrotic or apoptotic?

**Fig. 3:**
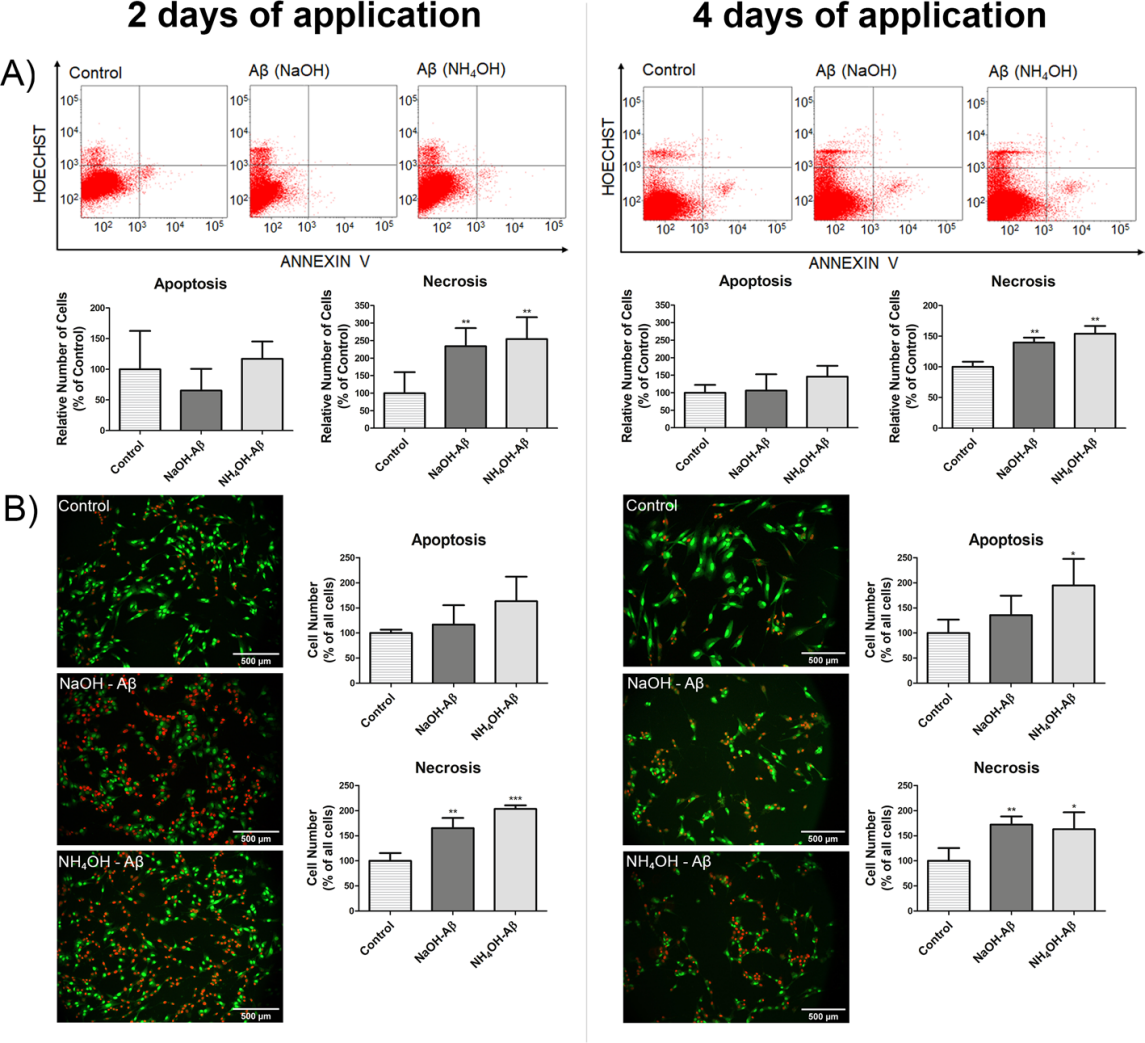
Assessment of apoptosis and necrosis. **A** Using flow cytometry, Annexin V/Hoechst staining revealed no significant change in apoptosis levels, but a notable increase in necrosis levels following 2 and 4 days of application of both NaOH-Aβ (*n* = 3, 139.67%,  = 0.00312; *n* = 5, 233.84%, *p* = 0.00879, respectively) and NH_4_OH-Aβ (*n* = 3, 154.081%, *p* = 0.00042; *n* = 5, 254.34%, *p* = 0.00327, respectively) preparations. **B** Results of the AO/PI assay (fluorescence microscopy) reflected these findings and showed an increased level of necrosis after 2 days (165,29%, *n* = 3, *p* = 0.00472 with NaOH-Aβ42; 203,39%, *n* = 3, *p* = 0.00041 with NH_4_OH-Aβ42) and 4 days (172,24%, *n* = 4, *p* = 0.00648 with NaOH-Aβ42; 163,02%, *n* = 4, *p* = 0.01511 with NH_4_OH-Aβ42). Additionally, NH_4_OH-Aβ42 treatment led to a significant elevation in the number of apoptotic cells (194.82%, *n* = 4, *p* = 0.01847). Statistical significance level versus untreated samples: *p ≤ 0.05, **p ≤ 0.01, ***p ≤ 0.001

We confirmed the toxicity of both Aβ42 preparations by measuring the level of p38, which was elevated after 4-day treatment (Supplementary Fig. S3). Activation of the ERK/p38 MAPK pathway is known to be connected with Aβ action [41, 44]. As we observed the toxicity of Aβ42 in our model, we asked the question of whether the cell death (CD) was necrotic or apoptotic. In search for the answer, we analyzed our samples by flow cytometry (FC). In accordance with the MTT and TB assay, an elevated number of dead cells was recorded. The Hoechst 33,258 dye is not able to enter healthy cells, but it can permeate disturbed membranes of necrotic cells [90, 95]. In our experiments, we observed an increase in the number of necrotic cells by ~ 40–150% during both the 2- and 4-day applications of NaOH- and NH_4_OH-Aβ preparations. On the other hand, annexin labeling showed no apoptosis to be underway in both preparations and in both time courses. However, in the 4-day application of the NH_4_OH-Aβ42, the number of apoptotic cells nonsignificantly increased, indicating the possible initiation of apoptosis (Fig. 3A).

The use of Acridine Orange and propidium iodide (AO/PI) as dyes, which are differentially incorporated into cells, also enables the monitoring of necrosis and apoptosis [91–93]. As shown in Fig. 3B, the results are similar to those from flow cytometry. We can again observe more than 150% increased number of necrotic cells (dark orange/red color) and, in the case of NH_4_OH-Aβ42 treated SH-SY5Y cells, also a slightly elevated number of apoptotic cells (bright-green nucleus with orange markings). We thus can conclude that, while both preparations of Aβ42 are toxic and cause necrosis of the differentiated SH-SY5Y cells, in 4-day application of NH_4_OH-Aβ42, apoptosis may also be involved, but only to a small extent.

### Apoptosis

**Fig. 4:**
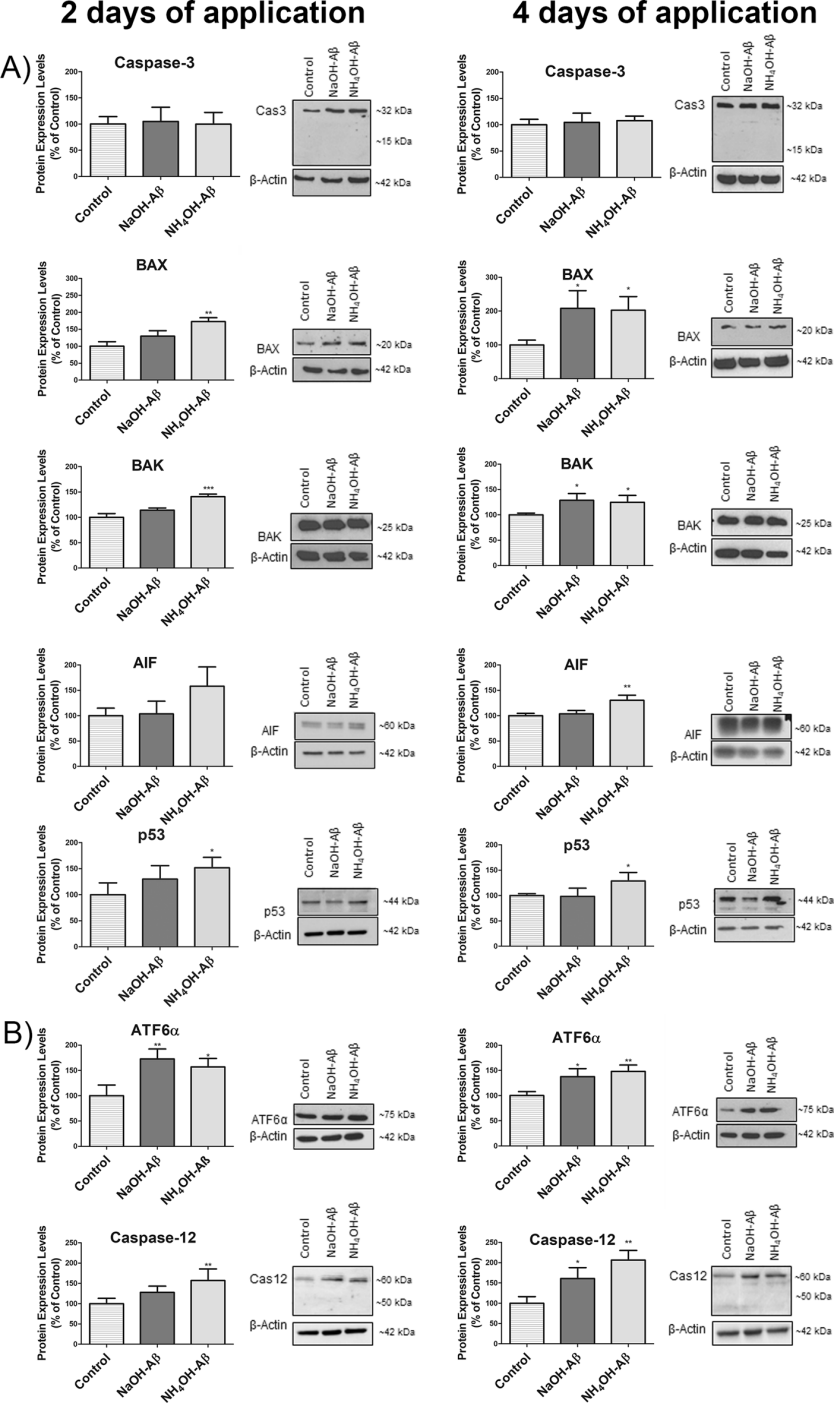
Differential modulation of apoptosis and ER stress response pathways by application of NaOH-Aβ42 and NH_4_OH-Aβ42. **A** Western blot analysis revealed changes in protein expression levels after 2 and 4 days of peptide treatment. BAX and BAK levels increased significantly after 2 days in NH_4_OH-Aβ42-treated samples (172.69%, *n* = 3, *p* = 0.00161 and 140.78%, *n* = 3, *p* = 0.00029, respectively), while after 4 days, elevation was observed in both NaOH-Aβ (208.11%, *n* = 3, *p* = 0.03325 and 128.61%, *n* = 3, *p* = 0.01048, respectively) and NH_4_OH-Aβ (202.70%, *n* = 3, *p* = 0.04082 and 124.78%, *n* = 3, *p* = 0.02408, respectively) samples. AIF levels significantly increased only after 4 days of NH_4_OH-Aβ treatment (130.18%, *n* = 3, *p* = 0.00651), along with elevated p53 expression at both time points (151.78%, *n* = 5, *p* = 0.01013 after 2 days and 128.80%, *n* = 4, *p* = 0.03613 after 4 days). **B** ATF6α levels showed a consistent elevation in all preparations, with a significant increase observed after 2 days with NaOH-Aβ42 (172.73%, *n* = 3, *p* = 0.00849) and NH_4_OH-Aβ42 (156.94%, *n* = 3, *p* = 0.02558), and after 4 days with NaOH-Aβ42 (137.77%, *n* = 3, *p* = 0.02398) and NH_4_OH-Aβ42 (148.07%, *n* = 3, *p* = 0.00803). After 2 days, a significant increase in Cas12 levels was observed only with NH_4_OH-Aβ42 (156.96%, *n* = 3, *p* = 0.03186), while after 4 days of treatment, Cas12 levels increased markedly with both NaOH-Aβ42 (160.92%, *n* = 3, *p* = 0.03835) and NH_4_OH-Aβ42 (206.60%, *n* = 3, *p* = 0.00291). Statistical significance level versus untreated samples: *p ≤ 0.05, **p ≤ 0.01, ***p ≤ 0.001

Previous experiments were aimed at later stages of apoptosis connected with PM remodeling. To monitor apoptosis in more detail, we detected selected apoptotic markers connected with different stages of the process. As a hallmark of apoptosis, the activation of executioner caspases including Cas3 is a characteristic event. However, in all our experiments, no Cas3 level elevation or cleavage was detected (Fig. 4A). This result is in accordance with data from FC and AO/PI measurements, where no apoptosis was detected except in the NH_4_OH-Aβ42 4-day group, where a small portion of apoptotic cells was present.

In caspase-independent apoptosis [1, 28–32], BAX and BAK proteins self-organize into the MOMP through which proapoptotic elements release mitochondria, including the AIF. In our model, 2-day treatment led to an elevation of BAX and BAK only in the NH_4_OH-Aβ42 preparation, to 172.69% and 140.78%, respectively. However, after 4 days of Aβ42 application, they were elevated in both the NaOH- and NH_4_OH-Aβ42 samples (more than 200% in BAX and more than 120% in BAK). On the other hand, AIF significantly increased only in the 4-day NH_4_OH-Aβ42 application, for 130%, where p53, a transcription factor associated with apoptosis activation [106–108] was also elevated (after 2 days 151% and after 4 days 128%). As shown in Fig. 4A, p53 increased also in the 2-day NH_4_OH-Aβ42 application. Based on our data, we can conclude that, although apoptosis is not the main pathway of CD in our experiments, it appears that initial activation began, at least in the 4-day NH_4_OH-Aβ42 application.

ER stress may serve as one of many entry sites to CD. We analyzed two important markers of ER stress, ATF6α and Cas12. Figure 4B shows that the amount of ATF6α increased after 2 days of treatment in both preparations, to more than 140%. Similar data were obtained for 4 days of application. However, at 2 days, the amount of Cas12 increased (156%) only after NH_4_OH-Aβ42 treatment, whereas no significant difference was detected after incubation with NaOH-Aβ42. These data indicate ER-induced apoptosis, as also observed in the SH-SY5Y model [109, 110]. Thus, we can assume that both Aβ species induced damage to the ER function to a similar extent.

### Reactive oxygen species

**Fig. 5:**
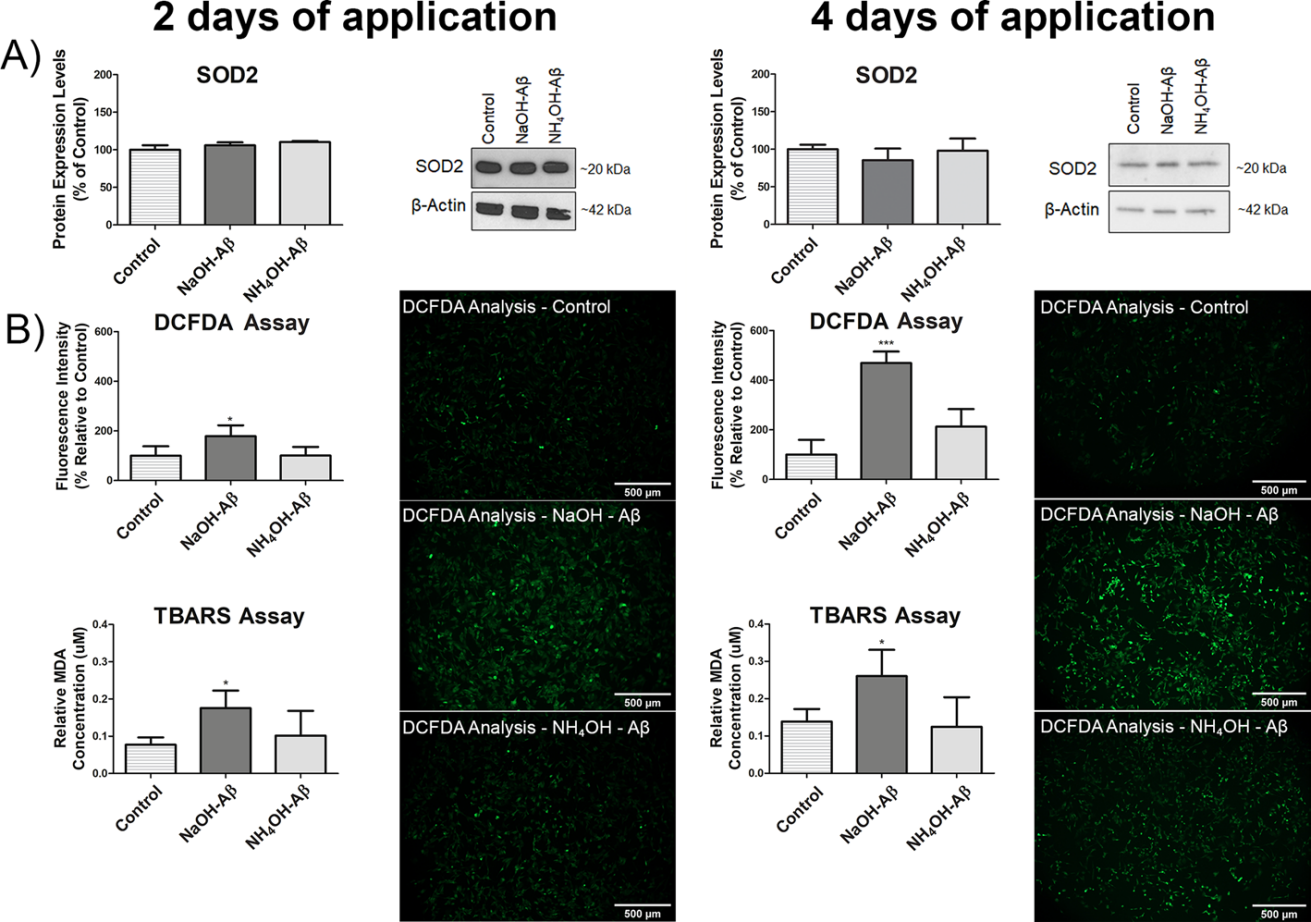
Assessment of oxidative stress markers in response to NaOH-Aβ42 and NH_4_OH-Aβ42 treatment. **A** SOD levels remained unchanged following 2- and 4-day application of both peptide preparations (*n* = 3). **B** DCFDA and TBARS assay to evaluate oxidative stress. DCFDA assay reveals a significant increase in fluorescence signal only in NaOH-Aβ42-treated cells after 2 days (178.57%, *n* = 4, *p* = 0.0399) and 4 days (469.10%, *n* = 3, *p* = 0.00018) of exposure, while no elevation is observed in NH_4_OH-Aβ42-treated cells. TBARS assay demonstrates a consistent increase in malondialdehyde (MDA) levels, indicative of lipid peroxidation, with a 227.18% increase (*n* = 4, *p* = 0.04363) after 2 days and a 188.15% increase (*n* = 3, *p* = 0.02778) after 4 days of treatment. Statistical significance level versus untreated samples: *p ≤ 0.05, **p ≤ 0.01, ***p ≤ 0.001)

It is well known that Aβ42 action is related to increased production of reactive oxygen species (ROS). We thus measured ROS-induced damage by determining levels of key protective enzymes superoxide dismutases (SOD), mainly cytosolic SOD1 and mitochondrial SOD2 (Fig. 5A). Although these enzymes represent the main barrier against oxidative stress connected with ROS, we did not detect any alteration of SOD in any of our experimental settings.

For monitoring ROS-induced damage, we used two independent methods, viz. DCF-DA and TBARS assays. Our results from the DCF-DA assay show significantly increased fluorescence signal only in NaOH-Aβ42-treated cells, in both 2- (178% increase) and 4-day (469% increase) exposure, whereas no elevation was observed in the NH_4_OH-Aβ42 samples (Fig. 5B). This indicates a difference in the action of individual Aβ42 species in relation to oxidative stress. We thus wanted to confirm our result by a different method, so we monitored the level of lipid peroxidation, which is a characteristic feature of ROS interaction with membrane lipids. In the TBARS assay, TBA reacts with malondialdehyde (MDA), a cleavage product of lipids produced by ROS in a membrane environment. The reaction product TBARS yields a red–pink color that can be measured spectrophotometrically at 532 nm [85]. As shown in Fig. 5C, we again monitored more than a 150% increase in MDA level, only in NaOH-Aβ42-treated cells. We can thus conclude that, in our experiments, only NaOH-Aβ42 induced ROS damage, but to an extent that does not activate the anti-ROS protecting system based on SODs.

### Effect on mitochondria

**Fig. 6:**
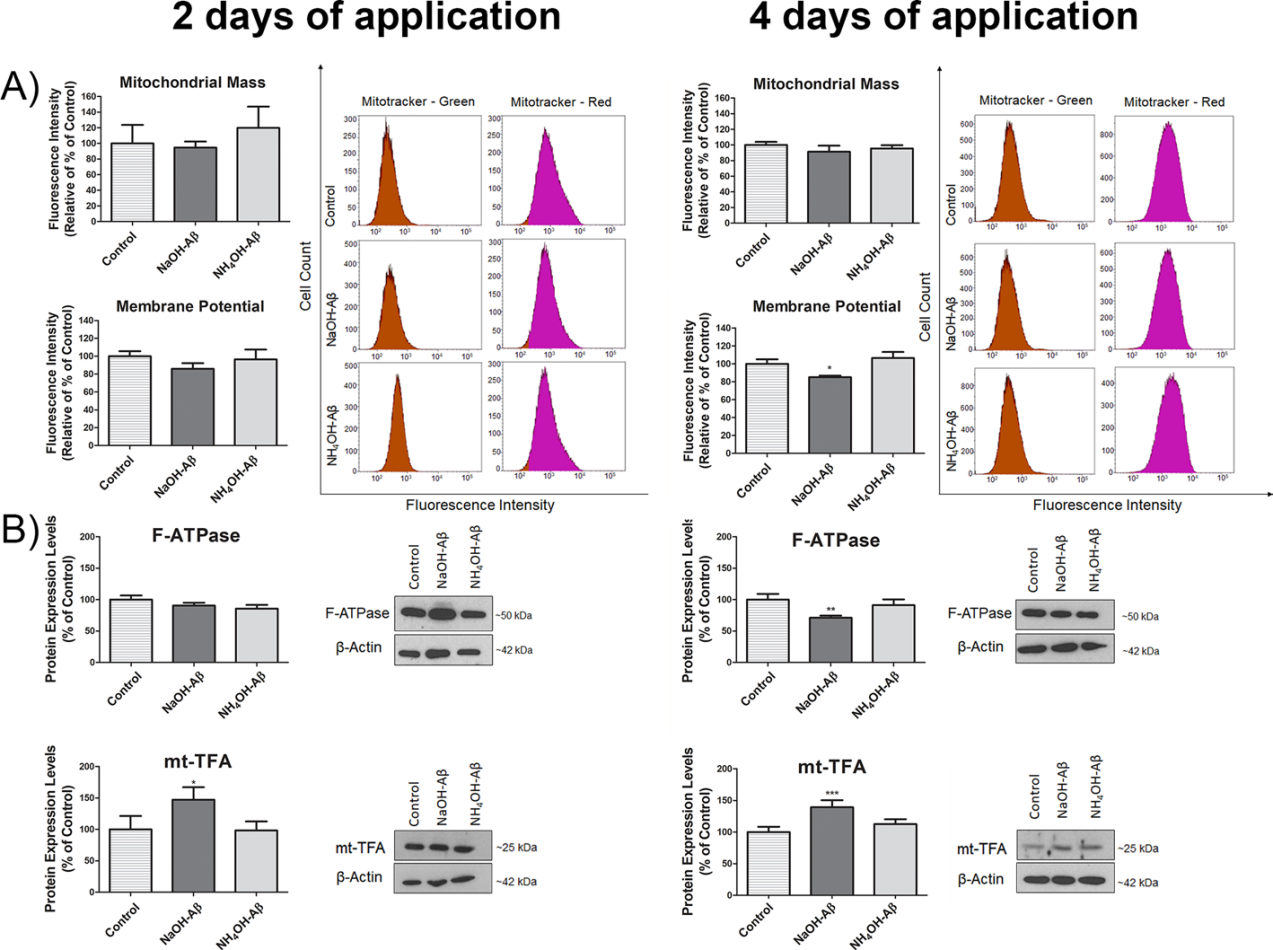
Analysis of mitochondrial function and protein expression in response to NaOH-Aβ42 and NH_4_OH-Aβ42 treatment. **A** MitoTracker analysis showed a decrease in mitochondrial membrane potential in cells exposed to NaOH-Aβ42 after 4 days (85.01%, *n* = 3, *p* = 0.02525), although mitochondrial mass had not changed. **B** The expression levels of F-ATPase showed a significant decrease in NaOH-Aβ42-treated cells after 4 days (71.14%, *n* = 3, *p* = 0.00847), while they remained unchanged when NH_4_OH-Aβ42 was applied. Conversely, mt-TFA levels increased only in NaOH-Aβ42 experiments after 2 days (139.18%, *n* = 4, *p* = 0.01517) and 4 days (139.18%, *n* = 4, *p* = 0.00054) of amyloid exposure. Statistical significance level versus untreated samples: *p ≤ 0.05, **p ≤ 0.01, ***p ≤ 0.001

As ROS are produced mainly in mitochondria, these organelles are exposed to their action first. Thus, we studied changes in the mitochondrial membrane potential, the level of the main mitochondrial enzyme, the F-ATPase, and the production of mt-TFA (TFAM), all of which may indicate disruption of mitochondrial homeostasis. We measured mitochondrial mass and membrane potential with MitoTracker™ red and MitoTracker™ green, respectively (Fig. 6). Although no alteration in mitochondrial mass was observed, there was a decrease in the mitochondrial membrane potential to 85% in NaOH-Aβ42-exposed cells, which was apparent in 4-day Aβ treatment. This alteration corresponded well to immunoblot analysis of the expression of both F-ATPase and mt-TFA (Fig. 6B). While the amount of F-ATPase significantly decreased in NaOH-Aβ42-treated SH-SY5Y cells after 4 days of exposure, to 71.14%, it remained unchanged in NH_4_OH-Aβ42 application. The reverse was monitored for mt-TFA level, which was increased only in NaOH-Aβ-treated cells, with a more conspicuous effect after 4 days of amyloid action (~ 140%). Although the membrane potential was only slightly decreased and no reduction in mitochondrial mass occurred, the decrease of the F-ATPase and elevation of mt-TFA indicates hampering of mitochondrial activity after NaOH-Aβ42 action. On the other hand, the NH_4_OH-Aβ42 seemed not to alter any of the monitored parameters of mitochondrial function.

### Calpain activity

**Fig. 7:**
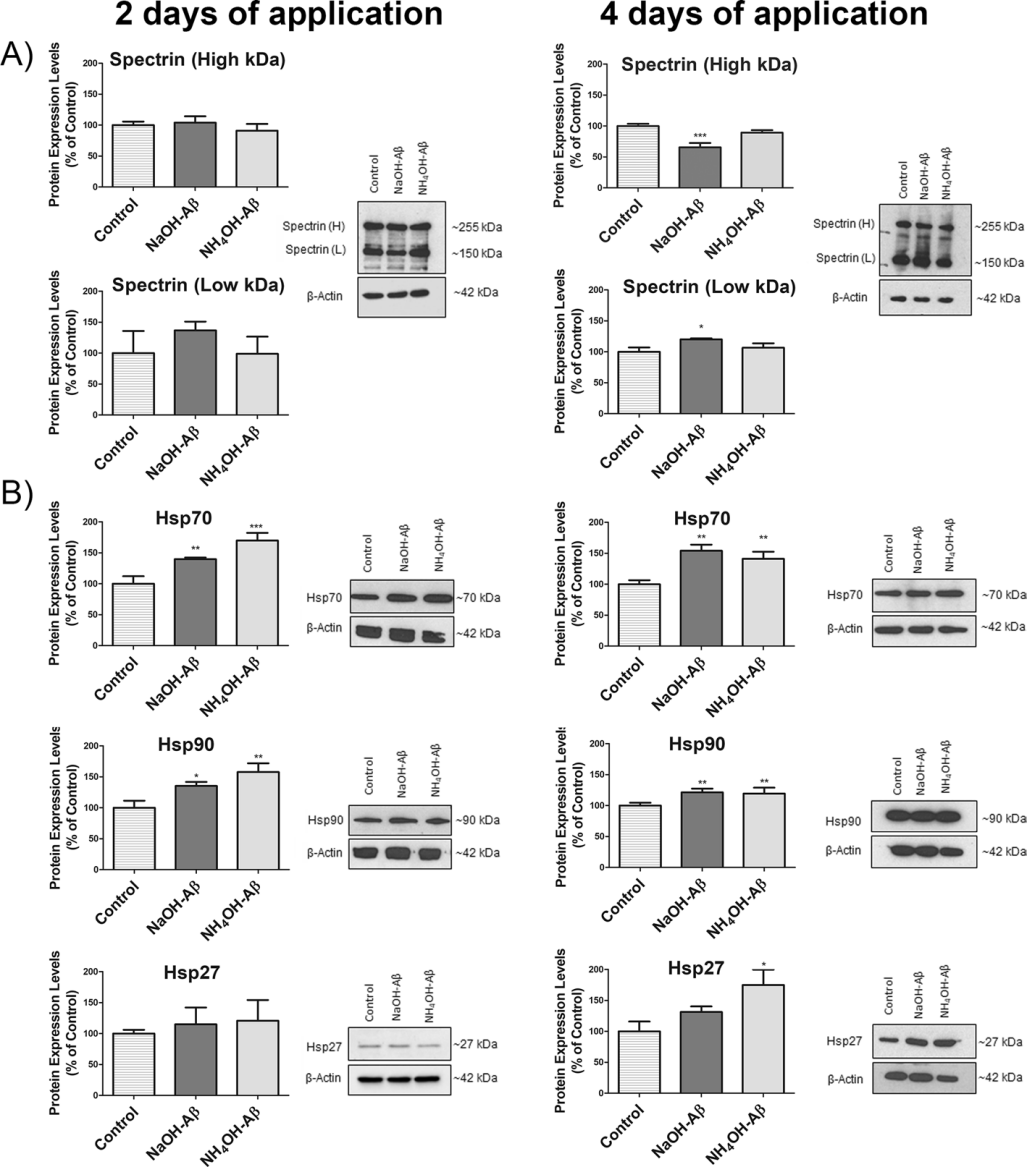
Analysis of calpain activity and heat shock protein (Hsp) expression in response to NaOH-Aβ42 and NH_4_OH-Aβ42 treatment. **A** Enhanced calpain activity was evident only in cells treated with NaOH-Aβ42 for 4 days, as indicated by the reduction of the full-length spectrin form (~ 255 kDa) to 65.64% (*n* = 3, *p* = 0.00039) and parallel elevation of a lower-weight spectrin form (~ 150 kDa) to 120.15% (*n* = 3, *p* = 0.01293). **B **The levels of Hsp70 and Hsp90 showed a consistent increase in response to both NaOH-Aβ42 and NH_4_OH-Aβ42 treatments, observed in both 2- and 4-day applications (Hsp70: after 2 days of exposure to NaOH-Aβ42 139.73%, *n* = 3, *p* = 0.00735, and 169.86%, *n* = 3, *p* = 0.00038 after NH_4_OH-Aβ42 applications, after 4 days of exposure 154.15%, *n* = 3, *p* = 0.00104 after NaOH-Aβ42, and 141.05%, *n* = 3, *p* = 0.00439 after NH_4_OH-Aβ42 application; Hsp90: after 2 days of exposure 135.22%, *n* = 3, *p* = 0.01930 after NaOH-Aβ42 and 157.83%, *n* = 3, *p* = 0.00172 after NH_4_OH-application, after 4 days of exposure 121,41%, *n* = 3, *p* = 0.00489 after NaOH-Aβ42 and 119.44%, *n* = 3, *p* = 0.00877 after NH_4_OH-Aβ42 application). Hsp27, however, was significantly increased only after 4 days of NH_4_OH-Aβ42 exposure, indicating a specific protective response (174.89%, *n* = 3, *p* = 0.00513). Statistical significance level versus untreated samples: *p ≤ 0.05, **p ≤ 0.01, ***p ≤ 0.001

In the previous experiments, we observed disruptions of ER and mitochondria, especially in SH-SY5Y cells exposed to NaOH-Aβ42. Both these organelles serve as the storage system of intracellular calcium ions. The alterations in ER/mitochondria integrity may thus indicate dyshomeostasis of cytosolic Ca^2+^ concentration. In our study, we concentrated on the long-term effects of Aβ treatment, so we analyzed the activity of Ca^2+^-dependent protease calpain, whose activation is often connected with Aβ action (reviewed in Refs. [1, 111]). Calpain activity is measurable by monitoring the cleavage of spectrin, which serves as a calpain substrate [111, 112]. Our immunoblot analysis showed enhanced spectrin cleavage only in NaOH-Aβ42 4-day experiments, which was measured as a reduction of full-length spectrin (~ 255 kDa) to 65.64% and parallel elevation (120.15%) of lower-kDa spectrin form (~ 150 kDa) (Fig. 7A). We did not analyze other calcium-dependent events as we only wanted to consider the main aspects of the relatively long-term action of different Aβ preparations. In our experiments, we could thus see that calpain, as a marker of elevated cytosolic Ca^2+^ level, was activated only by NaOH-Aβ42 but not by NH_4_OH-Aβ42 species.

### Heat shock proteins

As we could observe cellular stress on several levels, we may suppose a systemic answer that includes the action of heat shock proteins (Hsp). We analyzed levels of large Hsp (Hsp70 and Hsp90) that serve as molecular chaperons defending cells against misfolded and aggregated proteins [113]. In our experimental system, both Aβ42 preparations caused elevation of large Hsp levels, in all 2- and 4-day Aβ applications (Fig. 7B). Although we could not see any difference in Hsp70 and Hsp90 content between NaOH-Aβ42- and NH_4_OH-Aβ42-treated cells, the reverse was observed for small heat shock protein Hsp27. Here, only after 4-day exposure to NH_4_OH-Aβ42, SH-SY5Y cells increased production of this protective protein to 174% (Fig. 7B). This result indicates that both preparations of Aβ activated Hsp associated with response to misfolded/aggregated proteins but only NH_4_OH-Aβ42 added also protection that is based on the small Hsp27.

### Necroptosis and other cell death pathways

**Fig. 8:**
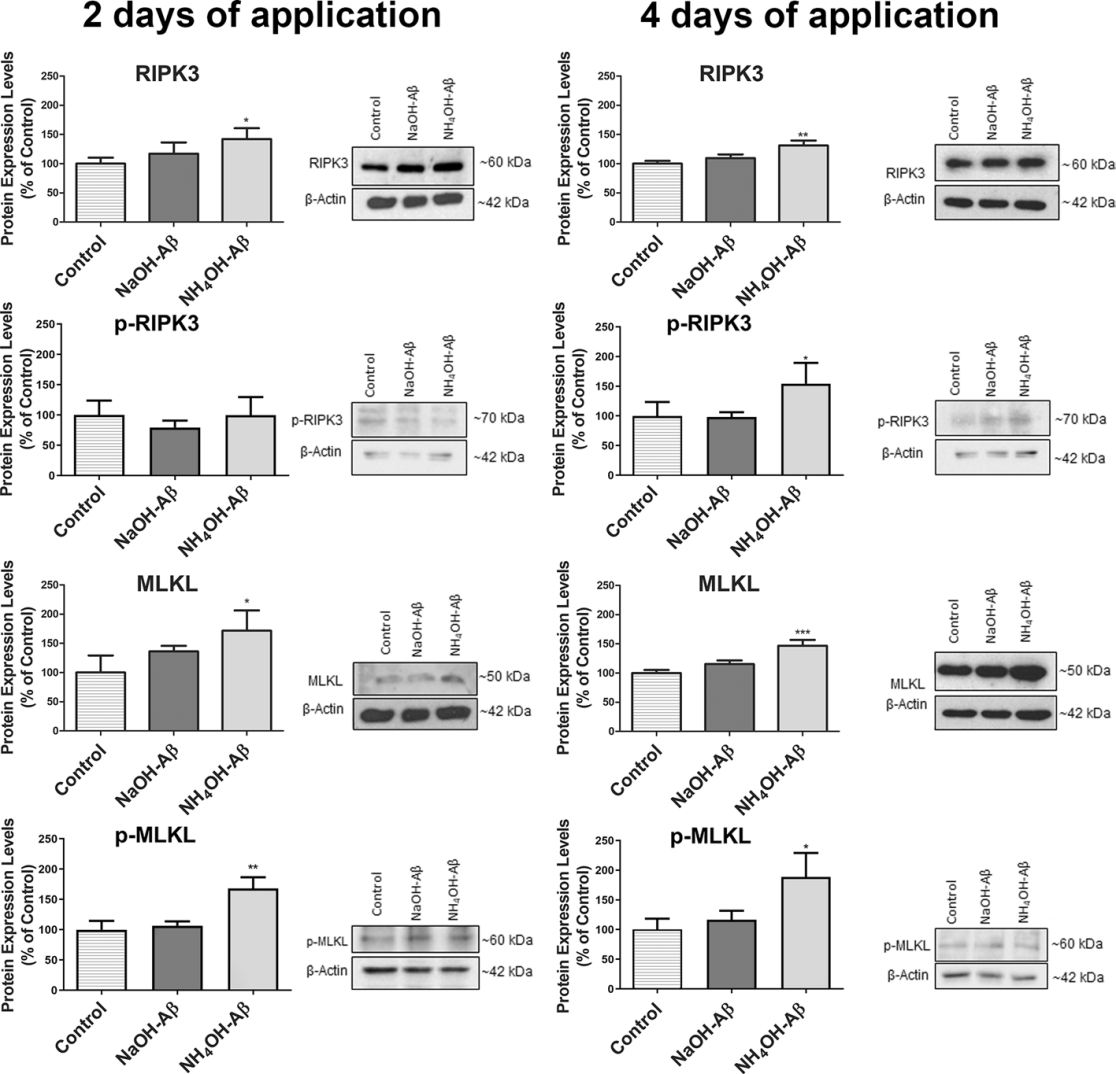
Quantification of necroptosis markers RIPK3 and MLKL and their phosphorylation in response to NaOH-Aβ42 and NH_4_OH-Aβ42 application. Treatment with NH_4_OH-Aβ42 for 2 days led to a significant increase in the levels of receptor-interacting protein kinase 3 (RIPK3, 142.23%, *n* = 3, *p* = 0.04589), mixed lineage kinase domain-like pseudokinase (MLKL, 171.49%, *n* = 3, *p* = 0.03958), and phosphorylated-MLKL (168.28%, *n* = 3, *p* = 0.00241). A more pronounced effect was observed after 4 days of NH_4_OH-Aβ42 exposure, with a significant increase in RIPK3 (131.18%, *n* = 3, *p* = 0.00319), MLKL (146.77%, *n* = 3, *p* = 0.00513), phosphorylated-RIPK3 (154, 35%, *n* = 4, *p* = 0.03047), and phosphorylated-MLKL (188.54%, *n* = 3, *p* = 0.01676). Statistical significance level versus untreated samples: *p ≤ 0.05, **p ≤ 0.01, ***p ≤ 0.001

Next, we focused our attention on necroptosis, programmed cell necrosis, which is also associated with AD [46, 114, 115]. In necroptosis, a necrosome complex from RIPK1 and RIPK3 is formed that leads to membrane disruption by MLKL [26, 45–48]. RIPK3 and MLKL as well as their phosphorylated forms may serve as markers of necroptosis [45, 48, 116]. In contrast to ROS-induced damage, we observed elevation of nonphosphorylated RIPK3 and MLKL only in NH_4_OH-Aβ42-exposed SH-SY5Y cells (Fig. 8). There was a clear effect in 2 days of Aβ treatment, but it was more significant in 4-day NH_4_OH-Aβ42 exposure. Analysis of RIPK3 and MLKL phosphorylation also indicated more than 170% elevation of these activated participants of necroptosis. On the other hand, no alterations in necroptosis markers were connected to NaOH-Aβ42 action (Fig. 8).

We were also interested in finding out whether other CD pathways might be involved in mediating the effects of Aβ. Measurements of PARP protein levels (a marker of parthanatos) and gasdermin D cleavage (a marker of pyroptosis) did not indicate alterations in these processes. Our results regarding autophagy were controversial, and this topic will be the subject of further research.

### Role of GM1

**Fig. 9:**
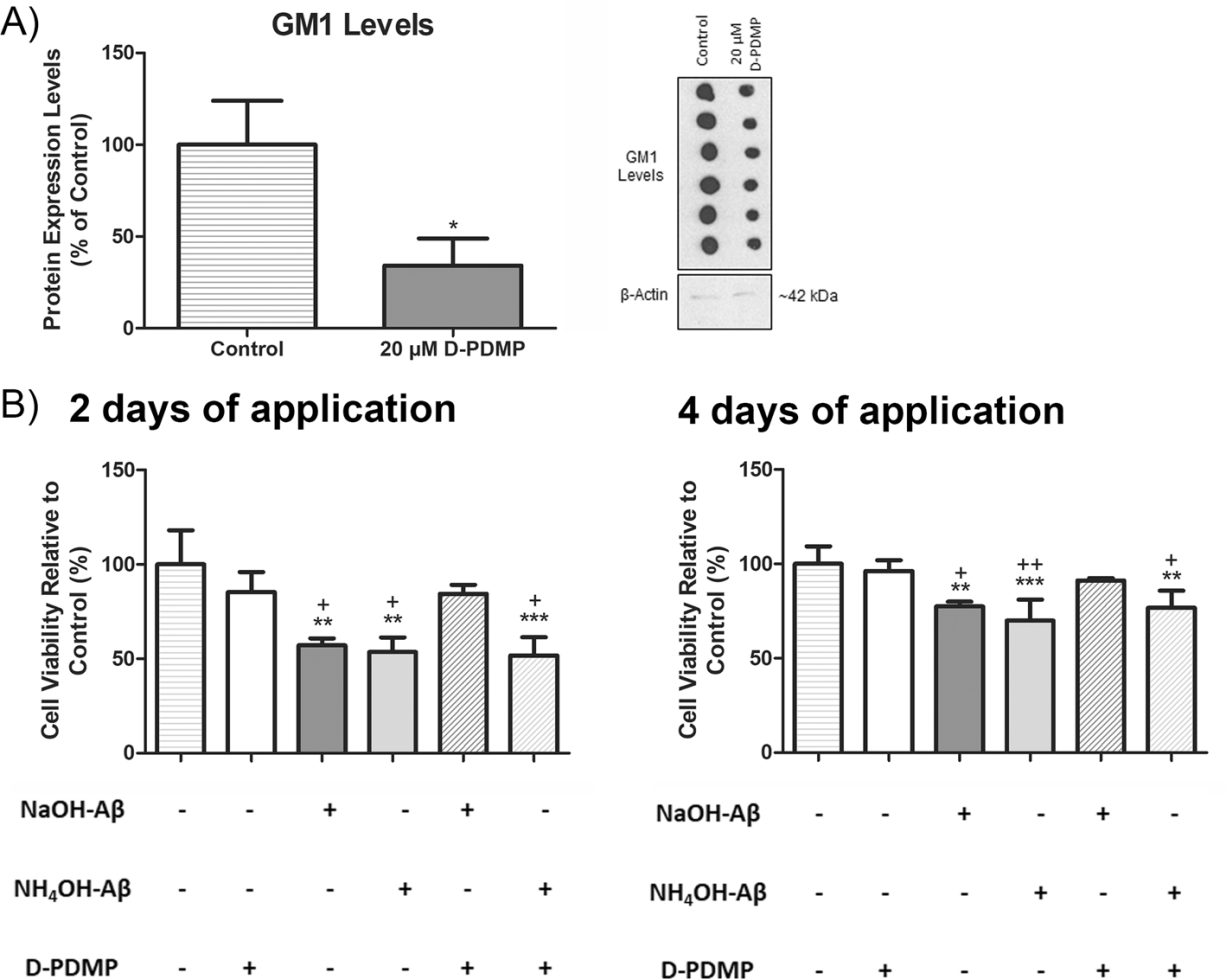
Modulation of GM1 levels and its impact on cell viability in response to Aβ42 peptides. **A** Treatment with 20 μM D-PDMP for 2 days resulted in a reduction of GM1 to 34.15% (*n* = 3, *p* = 0.0154); representative dot blot shown on the right. **B** Cells with decreased GM1 levels (D-PDMP+) did not show decreased viability even after 2 and 4 days of NaOH-Aβ42 application. However, cell viability was reduced after NaOH-Aβ42 action with normal GM1 level (D-PDMP−). On the other hand, NH_4_OH-Aβ42-treated cells showed unchanged Aβ toxicity even in the presence of D-PDMP (reduced GM1 level) (*n* = 3–4). Statistical significance level versus untreated samples (pluses) or samples treated only with D-PDMP (asterisks): *^/+^*p* ≤ 0.05, **^/++^*p* ≤ 0.01, ***^/+++^*p* ≤ 0.001

It is possible that NaOH-Aβ42 and NH_4_OH-Aβ42 may differ not only in their effect but also in their mechanism of entry into the cell. It is known that Aβ interactions with membrane lipid ganglioside GM1 may affect peptide aggregation, structure, and toxicity (reviewed in Ref. [22]). Using an inhibitor of ganglioside synthesis, D-PDMP [8, 20], we decreased the amount of membrane GM1 to 34.15% via the application of 20 μM D-PDMP for 2 days (Fig. 9A). The reduction of GM1 content by D-PDMP was not toxic to the differentiated cells (Supplementary Fig. S2), but it affected the Aβ action. Under D-PDMP-induced reduction of GM1, the NaOH-Aβ42 effect was diminished, and we did not detect any change in cell viability (Fig. 9). However, a similar reduction of GM1 did not affect the Aβ toxicity of NH_4_OH-Aβ42, where the viability was decreased to at least 75%, similarly to that with normal GM1 levels (Fig. 9).

## Discussion

The main purpose of the present study was to investigate the mechanisms responsible for cell death (CD) mediated by Aβ42. Among CD pathways that often may occur simultaneously, apoptosis, necroptosis, pyroptosis, altered autophagy and mitophagy, lysosomal destruction as well as ferroptosis and ROS action or mitochondrial disruption have been described to be associated with AD [16, 30, 32–37, 39–44, 46, 49, 50, 54, 55, 63, 67, 69, 75, 84, 100, 102–105, 114, 115, 117–120]. In our study, we focused on the effect of two distinct Aβ42 oligomer species: NaOH-Aβ42 and NH_4_OH-Aβ42. We used differentiated SH-SY5Y neuroblastoma cells that represent a neural-like model suitable for measuring toxicity induced by various substances, including Aβ42 [76, 84, 99, 100]. As the time course of Aβ application may play a role, we compared 2- and 4-day treatments. Although no significant difference in the size of both Aβ42 aggregates was found, they differed fundamentally in the type of CD they caused.

We prepared Aβ oligomers according to the protocols using NaOH or NH_4_OH for amyloid solubilization [73–76]. On the basis of DLS and AFM analyses, we do not expect strong differences in size/form and shape of both Aβ preparations, but it is possible that the size distribution in each population differs slightly. Although some fibrillar forms were observed, most of the particles had circular symmetry and a size corresponding to < 20 monomers. On the immunoblot, we could see bands corresponding to Aβ42 tetramers and to a much lesser extent also trimers. Antibodies against Aβ oligomers were not able to recognize these smaller forms, but they distinguished two larger aggregates of ~ 64 kDa and ~ 57 kDa MW corresponding to oligomers composed of ~ 14 and ~ 12 monomers of Aβ42. Whatever the size of the oligomers in our preparations, we could not find any difference in the size and shape of NaOH-Aβ42 and NH_4_OH-Aβ42 aggregates. Thus, owing to their specific effects on cells, we suppose more subtle differences in tertiary or secondary structure. We can only speculate what could be behind this different action of both preparations. We can exclude heterogeneity of Aβ coming from different sources (we used only one type from Bachem, 107,761–42-2), which may also play a role in the final effect. One potential possibility could be the different chirality of the growing fibrils (initial seeds of fibrils), as revealed by vibrational circular dichroism for mature fibrils [121]. Moreover, the sensitivity of the chiral form to the surrounding environment of growing fibrils, e.g., pH [121, 122], is well documented. Although this appears to be a plausible explanation, it is very difficult to verify in the case of Aβ (unlike model proteins). It is very hard to characterize relatively small Aβ initial aggregates more closely owing to the possible emergence of artificial rearrangements during sample manipulations (e.g., concentrations needed for vibrational circular dichroism are extremely high in comparison with those used in our experiments).

Both Aβ preparations were toxic to differentiated SH-SY5Y cells to a similar extent. We also excluded the toxicity of DMSO and pure NH_4_OH (Supplementary Fig. S2), which seems to be highly improbable owing to the very low concentration, high volatility, and long-term incubation. Different concentrations of Aβ are used experimentally, from < 1 µM to > 10 µM [16, 37, 39, 41, 44, 54, 57, 69, 75, 76, 84, 100, 102, 103, 105, 118, 120]. However, the final effective concentration is hard to establish, as (1) the aggregation process reduces the concentration of Aβ formations, and (2) cells concentrate Aβ intracellularly up to > 100 µM [123]. In our experiments, we mainly used the 2.25 µM starting concentration of Aβ, but we are aware that, during the aggregation process, the concentration of macromolecular formations decreased ~ tenfold compared with the monomers, and that a small amount of Aβ was lost in the form of larger fibrillar structures during the filtration of Aβ before addition to the cell culture.

AD is a slow neurodegeneration process, hence longer Aβ incubation better corresponds to pathophysiological processes in the brain. In almost all our experiments, in comparison with 2-day application, the effects of Aβ were accentuated after 4 days of exposure, and some of them seemed to just be beginning. Thus, the short incubation times that are widely used [16, 37, 39, 41, 44, 54, 69, 75, 102, 103, 105] may conceal information about processes with slower or delayed progression.

Among the types of CD related to AD, apoptosis may play a significant role [30, 32, 39–41, 44, 75, 100, 102, 103, 105, 119]. We thus focused on determining the contribution of apoptosis to CD caused by our Aβ42 preparations. The results from FC and AO/PI assay consistently showed the toxic effect of both NaOH- Aβ42 and NH_4_OH-Aβ42 preparations (Fig. 3), but a weak contribution of apoptosis was observed only in the 4-day NH_4_OH-Aβ exposure. Apoptosis includes a cascade of events where different elements are sequentially activated, including the activation of executioner Cas3. In accordance with the results from FC and AO/PI, we could detect no alteration in both procaspase 3 and the activated (cleaved) form after Aβ42 treatment (Fig. 4). Nevertheless, caspase-independent apoptosis was characterized, being associated with the formation of MOMP from BAX or BAK, release of AIF and cytochrome c from mitochondria, and activation of transcription factor p53 [1, 28–32, 124, 125]. In a concentration- and time-dependent way, BAX elevation and antiapoptotic Bcl-XL reduction do not need to induce activation of caspases, but increase cell vulnerability to ROS damage, as was shown on cultivated neurons exposed to Aβ42 [28]. Thus, the caspase-independent mechanism of Aβ toxicity is mediated by BAX-dependent mitochondrial membrane permeabilization and release of ROS [28–30, 32].

In the experiments with NH_4_OH-Aβ42-treated cells, proapoptotic BAX, BAK, AIF, and p53 increased after 4 days of exposure, indicating apoptosis activation. Although BAX/BAK pore formation may also be started in NaOH-Aβ42 preparation, where these proteins were elevated as well, no increase of AIF was observed, implying that full apoptosis was not elicited. However, we monitored only the total amounts of selected markers and not a change in cellular localization that would correspond to the release of AIF from mitochondria into the cytosol. We thus cannot exclude the shifts in cellular localizations that are not connected with alterations in total amounts. However, if they occurred, they were not accompanied by other apoptosis-related events. In accordance with FC and AO/PI, the results from WB analysis indicated that apoptosis was not a dominant process in Aβ-induced CD in the SH-SY5Y cell line. However, in NH_4_OH-Aβ42 action, caspase-independent apoptosis may be involved, as key markers of the initial phase of apoptosis seemed to be activated. The reluctance for apoptosis in our model of differentiated neuroblastoma cells may reflect the fact that mature neurons are resistant to apoptosis more than young cells, as they must survive for tens of years of the organism’s life [126].

ER stress represents a mitochondria-independent entry into the apoptosis that is also related to Aβ-induced damage and is connected with activation of caspases 4/12 and Ca^2+^ release [2, 70, 107, 127, 128]. ATF6α is a transcription factor that reacts to ER damage and supports apoptosis at multiple levels, including p53 action [124–126, 129]. In our experiments, both preparations of Aβ42 induced elevation of ATF6α as well as Cas12, whose activation was monitored during ER stress in SH-SY5Y cells [109, 110]. Although we did not observe Cas12 cleavage, the elevation of Cas12 and ATF6α indicates ER stress that may lead to apoptosis [130]. We may thus conclude that ER damage is associated with the action of both NaOH-Aβ4 and NH_4_OH-Aβ42 amyloid preparations.

As Aβ is known to induce ROS-associated damage [34, 39–44, 105], levels of superoxide dismutase (SOD) may mirror activation of anti-ROS defense. SOD2 was found to be protective against Aβ action [131], but Aβ induced a decrease of SOD2 in mouse and human AD brains [132, 133]. Aβ was also shown to reduce the activity of SOD1 [134] and the brain level of SOD1 decreased in AD human subjects [135], which may correspond to increased ROS-induced stress [136]. On the other hand, Aβ oligomers stimulated SOD1 activity in cultured neurons and in human AD brain [137]. Hence, SOD upregulation may compensate for ROS-induced damage [136]. The controversy regarding SOD1/2 alterations in AD reflects insufficient data, the use of different brain tissues, and the influences of other factors, including tau pathology as well as the time course. Both SODs were reduced at 3 and 18 months but increased in the 12-month-old brain neocortex of mice model of AD, which may indicate a loss of antioxidant capacity in the later stadia of AD [138]. However, no alterations in the amounts of SODs were monitored in our experiments.

Although the protective action of SODs was not activated in our system, ROS production was enhanced. Production of intracellular ROS can be detected by the DCF-DA assay, where dichlorofluorescein (DCF) gives a fluorescence signal after ROS-mediated oxidation [42]. In the TBARS assay, the level of malondialdehyde (MDA) is measured by a reaction where the product TBARS may be evaluated spectrophotometrically [86]. Our results from both measurements clearly showed increased ROS production only in NaOH-Aβ42, and not in NH_4_OH-Aβ42-treated SH-SY5Y cells. The effect was observed in both 2- and 4-day experiments, which means a stable and relatively long-term oxidative stress. Higher ROS damage in NaOH-Aβ42-treated cells was surprising as ROS-induced deleterious processes are interconnected with apoptosis and our measurements of apoptosis indicated a stronger effect in NH_4_OH-Aβ42-exposed SH-SY5Y cells.

AD-related mitochondrial dysfunction induced by Aβ is associated with alterations in the mitochondrial membrane potential [6, 43, 69, 84, 120, 139]. Our data confirmed this effect, but only in the case of NaOH-Aβ42 action. Although no reduction in mitochondrial mass was observed, the key enzyme, F-ATPase (ATP-synthase) amount was lowered in NaOH-Aβ42-treated SH-SY5Y cells. F-ATPase was shown to be decreased in AD brains [140], and it serves as a substratum of ROS in AD [113, 141]. Our observation of decreased F-ATPase amount in NaOH-Aβ42-exposed cells corresponds to the elevation of oxidative stress measured by DCF-DA and TBARS assays.

Mt-TFA (TFAM) participates in the regulation of Ca^2+^ and ROS levels as it is a part of a protective system for mitochondrial DNA [105, 142–144]. Mitochondrial ROS may suppress mt-TFA expression [144, 145], and a decreased amount of mt-TFA was observed in human AD hippocampi and the M17 cellular model of AD [146]. On the other hand, mt-TFA elevation may serve as a compensation mechanism even toward Aβ42-induced ROS damage [142, 147]. The overexpression of mt-TFA attenuated Aβ-induced oxidative damage of mitochondrial DNA in SH-SY5Y cells [105]. In our experiments, mt-TFA was elevated only in NaOH-Aβ42-exposed cells, especially after 4 days of treatment. This indicates activation of anti-ROS defense at the level of mitochondria, although we did not monitor any effect on mitochondrial SOD2 level. Based on obtained data, we can conclude that NaOH-Aβ42, but not NH_4_OH-Aβ42, is connected with ROS-induced damage of mitochondrial function and at least partial activation of protective processes represented by elevation of mt-TFA.

Amyloid β may cause calcium dyshomeostasis by enhancing the Ca^2+^ permeability of plasma, ER, or mitochondrial membrane [1, 3, 6, 16, 43, 148, 149]. In AD, elevated intracellular Ca^2+^ level activates calpain protease, which may lead to CD [1, 111, 112]. Because we observed disruption of both main Ca^2+^ storage systems, ER and mitochondria, which was accentuated in NaOH-Aβ42 exposure, Ca^2+^ dyshomeostasis seemed highly probable. We thus analyzed calpain activity by measuring the cleavage of calpain substrate, spectrin [111, 112]. As the main aim of our study was a characterization of the long-term effects of Aβ42, calpain activation represented a system responding to prolonged Aβ treatment and our results showed that only in 4-day treatment with NaOH-Aβ42 preparation was spectrin cleavage increased. This indicates that Ca^2+^-dependent processes including calpain activation are associated with longer times of Aβ exposure, as we did not observe enhanced spectrin cleavage in 2-day experiments. We recorded ER stress (ATF6α and Cas12 elevations) in both Aβ preparations, but calpain activation and mitochondrial damage were apparent only in NaOH-Aβ42 exposures, and not in NH_4_OH-Aβ42 treatment. Thus, we can hypothesize that Ca^2+^ ions were released from mitochondria and not from ER, as only 4 days of NaOH-Aβ42 exposure led to calpain activation.

Among protective mechanisms activated by Aβ, Hsp may play a role. Small Hsp, including Hsp27 (HspB1), may protect cells against ROS and thus be elevated in AD. Hsp27 was shown to act antiapoptotically at several levels, mainly by interacting with BAK, BAX, p53, cytochrome c, PI3K/Akt, or caspases (reviewed in Ref. [108]), but also by reducing Aβ toxicity by affecting its quaternary structure [150, 151]. On the other hand, the large neuroprotective Hsp (including Hsp70) were lowered in AD brains (reviewed in Ref. [151]). However, in our experiments, the levels of Hsp70 and Hsp90 were increased, which may mean activation of protective system related to misfolded and aggregated proteins [113, 151–153]. Our data thus show that, although both Aβ preparations, NaOH-Aβ42 and NH_4_OH-Aβ42, activated protection against elevated misfolded/aggregated proteins based on Hsp70 and Hsp90, only in the case of NH_4_OH-Aβ42 exposures was antiapoptotic and anti-ROS protection mediated by Hsp27 also initiated.

Among other AD-associated CD mechanisms, necroptosis may play a role in neurodegeneration [46, 114, 115]. Necroptosis is initiated mainly when the activity of caspases is reduced, e.g., in mature neurons [126], where necroptosis may be the main actor in regulated CD [48]. Necroptosis is associated with creation of a multiprotein complex (necrosome) from receptor-interacting protein kinases (RIPK) 1 and 3, whose cooperation leads to phosphorylation and oligomerization of mixed lineage kinase domain-like pseudokinase (MLKL), bilayer permeabilization, and CD [26, 45–48]. Elevated and phosphorylated RIPK1 and MLKL were monitored in AD [36]. Necroptosis activation was observed also in neurons of human AD brains in relation to tau pathology or after tumor necrosis factor (TNF)-α secretion by microglia [46, 154, 155]. In a recent study, Aβ induced necroptosis even in the SH-SY5Y cell model [114].

In our experiments, only NH_4_OH-Aβ42 induced activation of necroptosis, which was measured as an increased amount and phosphorylation of both RIPK3 and MLKL. This result is somewhat surprising. On the basis of previous measurements, we supposed a necrotic process, but the activation of necroptosis in NH_4_OH-Aβ42-treated cells was not as expected. This is because necroptosis and apoptosis may behave as mutually competitive, especially owing to the distinct and mutually exclusive roles of RIPK1 in both processes [25, 26, 48, 65]. We can speculate that necroptosis is more likely to be activated in the earlier stages, while the apoptosis begins to emerge later, as we observed some apoptotic signals mainly after 4-day exposure to NH_4_OH-Aβ42 but still without activation of caspases. It would be very interesting to look at an even longer time scales of Aβ action.

Besides the time-course dependence, spatially directed response may also play a role. Different pathways may be activated in different cells, or even within different cell compartments. This was shown in the case of glutamate-mediated excitotoxicity, which was associated with apoptotic events in neuronal soma but with necroptosis in axons [156, 157]. We may thus hypothesize that the variability of the response to Aβ is connected to different intracellular contexts, the characteristics of particular Aβ forms, the time scale of Aβ action, and the membrane properties of a responsive cell.

It is well known that Aβ interacts with membrane receptors, including proteins and lipids. Among them, ganglioside GM1 was described as one of the most important Aβ partners [8, 20, 22, 158–163]. We thus compared the effects of NaOH-Aβ42 and NH_4_OH-Aβ42 on differentiated SH-SY5Y cell viability after decreasing the GM1 level with D-PDMP, the inhibitor of GM1 synthesis [20, 164]. In our experiments, D-PDMP was not toxic to cells and reduced GM1 levels effectively. What was interesting was the observation that reducing the GM1 level decreased the toxicity only of the NaOH-Aβ42 preparation, while the toxicity of NH_4_OH-Aβ42 remained unchanged. This indicates that NaOH-Aβ42 needs interaction with GM1 to mediate CD, while the toxicity of NH_4_OH-Aβ42 is GM1 independent. This result is especially interesting as various experimental groups have found distinct involvement of GM1, cholesterol, or sphingomyelin in Aβ-induced toxicity [17, 22, 23, 161, 165–167]. On the basis of observation, a part of this variability may be associated with distinct characteristics of particular Aβ species.

Finally, we also attempted to characterize other CD systems, including pyroptosis and autophagy, which are known to play a role in AD [26, 49, 50, 53–57, 63, 67, 117]. We did not record any alterations in levels and cleavage of PARP1 (marker of parthanatos) and gasdermin D (a marker of pyroptosis) (Supplementary Fig. 4). On the contrary, our results on autophagy remain ambiguous (not shown), and we will address the Aβ-induced alterations in autophagy in later studies.

## Conclusions

**Fig. 10:**
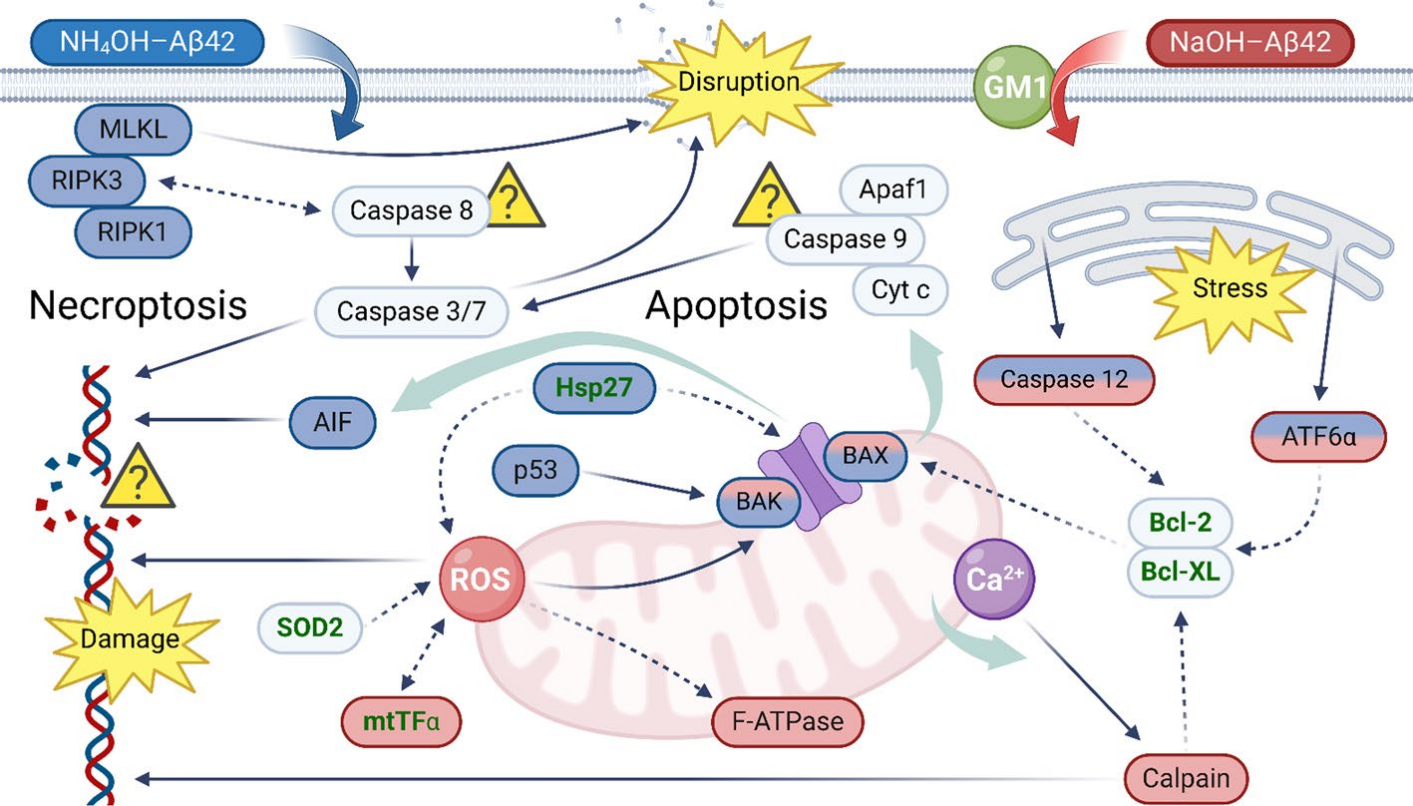
Diagram summarizing the presumed effect of the two Aβ42 preparations. NaOH-Aβ42 (red) mainly causes impairment of mitochondrial functions (increase of mt-TFα, decrease in F-ATPase, reduction in membrane potential) and induces damage by reactive oxygen species (ROS). At the same time, it activates calpain, which is related to the dysregulation of Ca^2+^ concentration. The toxicity of NaOH-Aβ42 depends on the amount of ganglioside GM1 in the membrane. In contrast, NH_4_OH-Aβ42 (blue) does not require GM1 and its action involves caspase-independent apoptosis-associated events and activation of necroptosis. In parallel, Hsp27-based protection against ROS is also activated. The amplification of endoplasmic reticulum stress appears to be common to both pathways, as well as an increase in BAX/BAK levels, indicating the formation of mitochondrial outer membrane pore. Prosurvival proteins are marked in green, and cell death-supporting elements in black. The solid arrows indicate positive effects, the dashed arrows show inhibitory effects, and the bold blue arrows indicate movement between compartments. We did not analyze DNA damage or shifts and levels of proapoptotic elements (caspase 8, 9, Apaf1 and cytochrome c, indicated by a question mark) as we assume that only initial steps of apoptosis take place. AIF, apoptosis-inducing factor; Cyt c, cytochrome c; MLKL, mixed lineage kinase domain-like pseudokinase; RIPK, receptor-interacting protein kinase; SOD, superoxide dismutase. (Created with BioRender.com)

Taken together, our observations show marked differences in the effects of two preparations of Aβ42, even though we could not see any distinction in their size and shape. Figure 10 indicates possible CD pathways initiated by NaOH-Aβ42 and NH_4_OH-Aβ42. The NaOH-Aβ42 preparation is connected to enhanced ROS production and associated disruption of mitochondrial integrity and function. On the other hand, the NH_4_OH-Aβ42 preparation is associated more with necroptosis and initiation of apoptosis, while mitochondria remain intact. Both amyloid species induce the first steps of apoptosis, and ER stress, and elevate Hsp-dependent protection against misfolded proteins. Only in NH_4_OH-Aβ42 action is the anti-ROS Hsp27 also elevated. We do not know whether the increased amount of Hsp27 or inability of this specific Aβ species to induce ROS-associated stress is responsible for the differences in mitochondrial damage of both preparations. As our measurements clearly showed necrosis to be present, the PM disruption occurred in all instances. Moreover, GM1 seems to play a distinct role in mechanisms of NaOH-Aβ42 and NH_4_OH-Aβ42 entry into the cell.

Our study has several limitations. First, we were not able to more closely characterize the structural differences between the two amyloid preparations. Second, we only outlined possible CD pathways activated by NaOH-Aβ42 and NH_4_OH-Aβ42, and we omitted many aspects of CD pathways. Third, the SH-SY5Y model is a very specific cellular system that in many ways does not correspond to brain neurons. In the future, we would like to further characterize the molecular structure of both Aβ preparations, to specify and extend the spectrum of analyzed intracellular processes associated with Aβ action, and finally to use another cellular model, preferably neural stem cells. It must be stressed here that different models inherently provide different data as Aβ-induced toxicity seems to be extremely sensitive to the context of its action, including membrane characteristics as well as the specific cytosolic environment. Thus, if even relatively small differences in the structure of a toxic amyloid preparation can play a role in its mechanism of action, it is not surprising that dozens or hundreds of studies have reached different results. It follows that all aspects related to the preparation of the AD model must be given the utmost attention. On the other hand, the different data from the brains of AD patients show that, even in vivo, not only is there variability in the toxic forms of Aβ but that the variability of the described damage fully corresponds to the diversity of tissues and cell types.

## Supplementary Information

Additional file 1.

## Data Availability

The data generated or analyzed during this study are included in this published article and its supplementary information files. All original WB and other direct outputs from measurements can be sent by the authors upon request.

## Abbreviations

Aβ42: Amyloid β42
AD: Alzheimer´s disease
AIF: Apoptosis inducing factor
AO: Acridine Orange
Cas: Caspase
CD: Cell death
DCF-DA: 2′,7′-Dichlorodihydrofluorescein diacetate
DLS: Dynamic light scattering
DMSO: Dimethyl sulfoxide
D-PDMP: *N*-[(1*R*,2)-2-hydroxy-1-(4-morpholinylmethyl)-2-phenylethyl]-decanamide
ER: Endoplasmic reticulum
FC: Flow cytometry
GM1: Monosialotetrahexosylganglioside
HFIP: 1,1,1,3,3,3-Hexafluoro-2-propanol
Hsp: Heat shock proteins
MDA: Malondialdehyde
MLKL: Mixed lineage kinase domain-like pseudokinase
MOMP: Mitochondrial outer membrane pore
MTT: 3-(4,5-Dimethylthiazol-2-yl)-2,5-diphenyltetrazolium bromide
mt-TFA (TFAM): Mitochondrial transcription factor A
MW: Molecular weight
PI: Propidium iodide
PM: Plasma membrane
RIPK: Receptor-interacting protein kinase
ROS: Reactive oxygen species
SOD: Superoxide dismutase
TBA: Thiobarbituric acid
TBARS: Thiobarbituric acid reactive substances
TCA: Trichloroacetic acid
WB: Western blot
