# Supplementary material for: Different amyloid β42 preparations induce different cell death pathways in the model of SH-SY5Y neuroblastoma cells
Source: Cell Mol Biol Lett. 2024 Nov 17;29:143. doi: 10.1186/s11658-024-00657-8 (PMC11572474; doi:10.1186/s11658-024-00657-8)
Supplement: Supplementary file 1 — Additional file 1. [file 11658_2024_657_MOESM1_ESM.pdf]

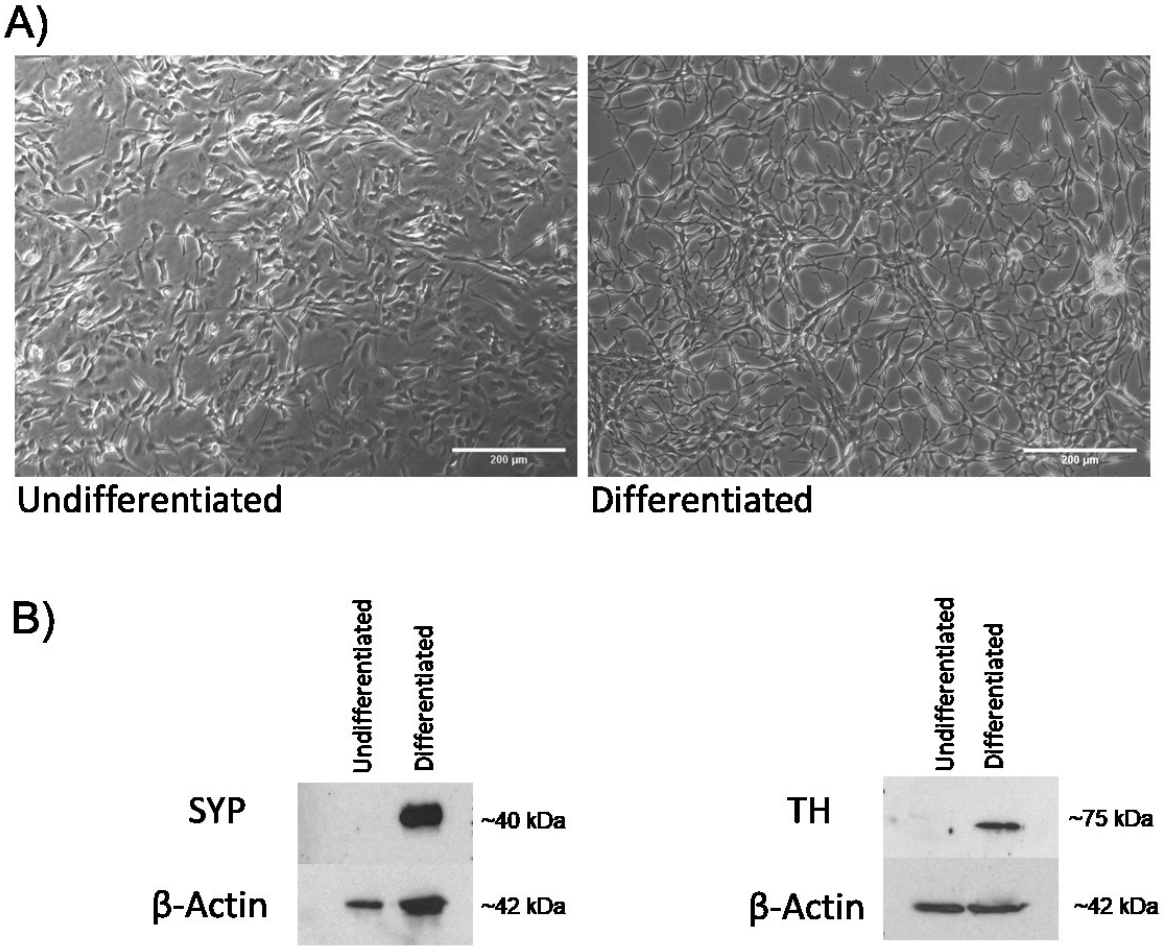

Supplementary Figure S1. Differentiation and characterization of SH-SY5Y cells. A) Representative 20X magnified images depict SH-SY5Y cells before and after 5 days of differentiation, illustrating morphological changes indicative of neuronal differentiation. B) Immunoblot analysis of synaptophysin (SYP) and tyrosine hydroxylase (TH) expression before and after differentiation highlights their importance as markers for neuronal differentiation. Synaptophysin is a synaptic vesicle protein involved in neurotransmitter release, while tyrosine hydroxylase is an enzyme involved in dopamine biosynthesis, both indicating successful neuronal differentiation [1, 2].

References

1. Khwanraj K, Phruksaniyom C, Madlah S, Dharmasaroja P. Differential Expression of Tyrosine Hydroxylase Protein and Apoptosis-Related Genes in Differentiated and Undifferentiated SH-SY5Y Neuroblastoma Cells Treated with MPP. Neurol Res Int 2015:734703.

2. Singh S, Somvanshi RK, Kumar U (2022) Somatostatin-Mediated Regulation of Retinoic Acid-Induced Differentiation of SH-SY5Y Cells: Neurotransmitters Phenotype Characterization. Biomedicines 2022;10:337.

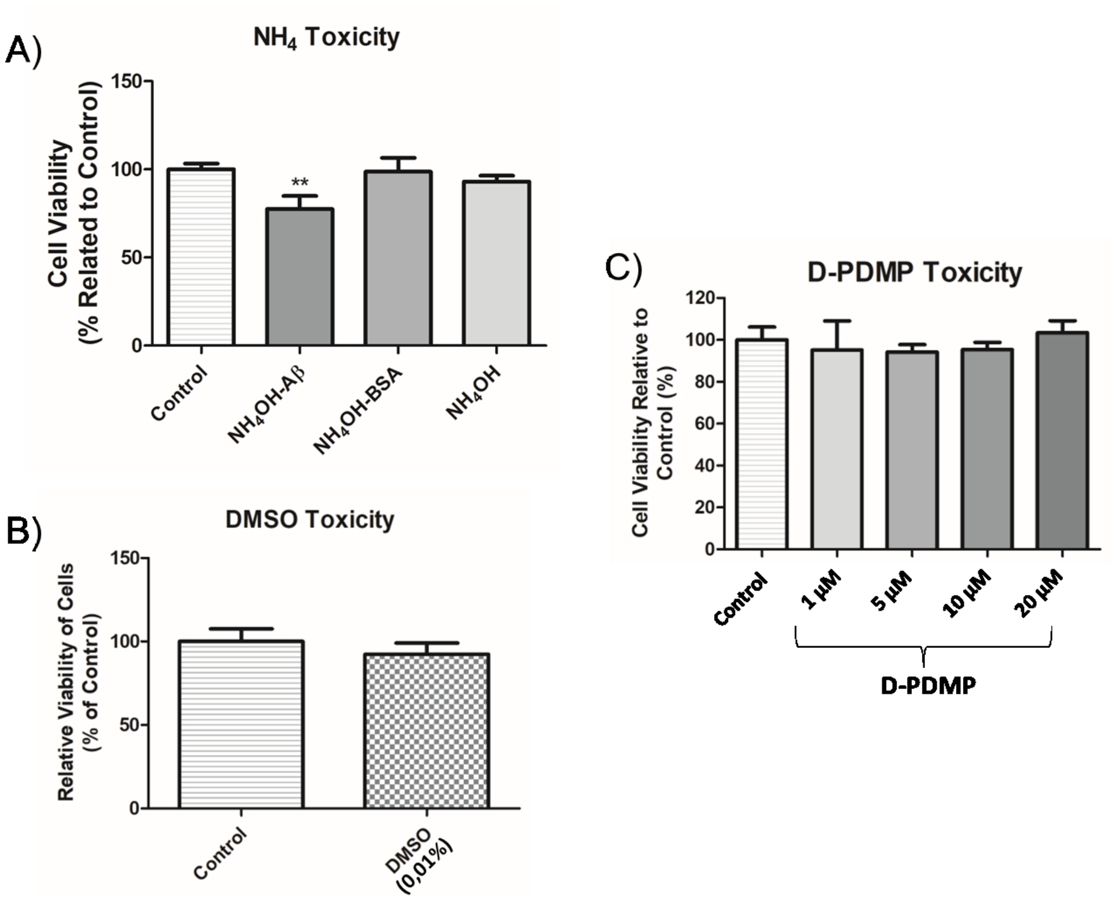

Supplementary Figure S2. Assessment of vehicle chemicals toxicity using MTT assay. A) To evaluate the potential toxicity of vehicle chemical NH_4OH, the same protocol utilized for Aβ oligomerization was followed with bovine serum albumin (BSA) and solely with NH_4OH. MTT test results demonstrated that NH_4OH did not exhibit toxicity to differentiated SH-SY5Y cells in the absence of Aβ. B) Assessment of 0.01% dimethyl sulfoxide (DMSO), and C) evaluation of D-PDMP toxicity. Results indicate no detrimental effects on cellular viability at the final concentration of any chemical tested. ($n=3$; **: statistical significance level versus untreated samples, $^{**}p \leq 0.01$).

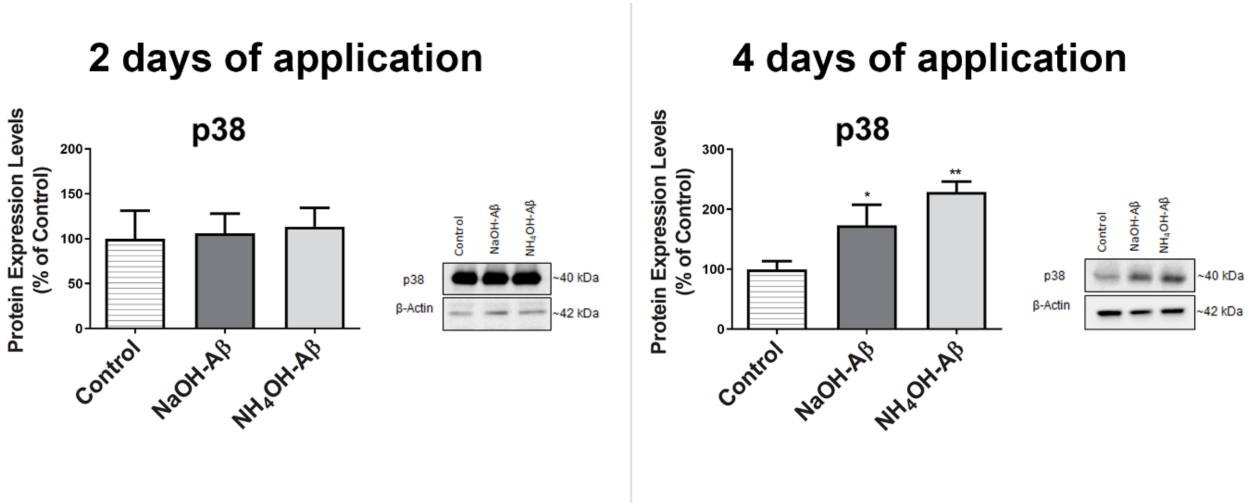

Supplementary Figure S3. Quantification of p38 protein response to NaOH-Aβ42 and NH_4OH-Aβ42 treatments. After 2 days of application there was no significant change in levels of p38. In contrast, after 4 days of application, a significant increase was detected in both NaOH-Aβ42 (173.11%, n=3, p=0.02116) and NH_4OH-Aβ42 (228.98%, n=3, p=0.00132) treatments (*, **: statistical significance level versus untreated samples, *p ≤ 0.05, **p ≤ 0.01).

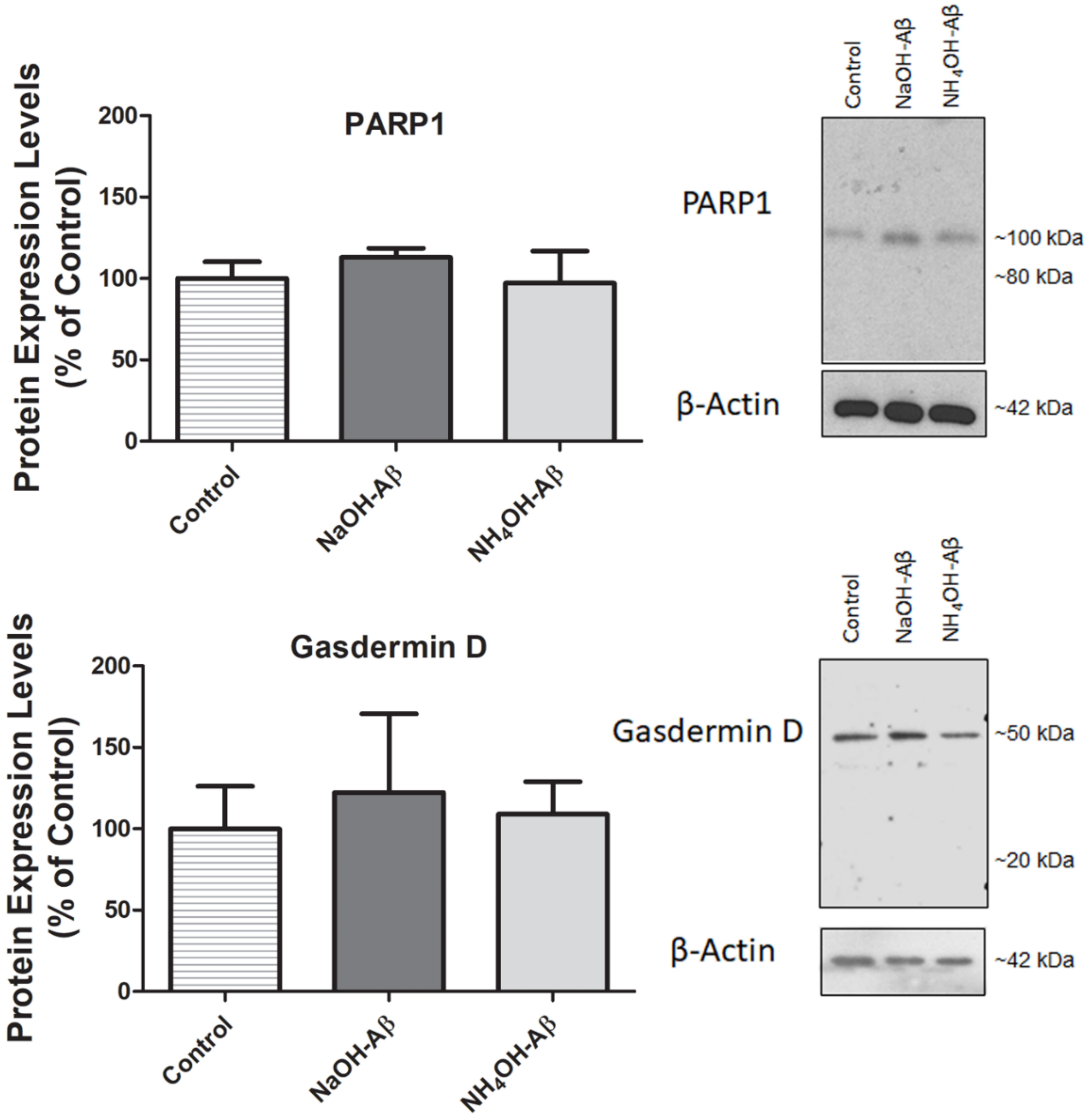

Supplementary Figure S4. Effect of Aβ42 peptides on parthanatos and pyroptosis in SH-SY5Y cells. Using western blot analysis, no alterations were observed in the levels and cleavage of PARP1 (marker of parthanatos) and gasdermin D (marker of pyroptosis) after 4 days of application of NaOH-Aβ42 or NH_4OH-Aβ42 (n=3).
